# Cost‐Effectiveness of CT Colonography Under Real‐World Colorectal Cancer Screening Adherence for Black and White Populations

**DOI:** 10.1002/cam4.71290

**Published:** 2025-11-12

**Authors:** Szu‐Yu Zoe Kao, Maria X. Sanmartin, Judy Yee, Kevin J. Chang, Courtney A. Moreno, Cecelia Brewington, David H. Bruining, Eric W. Christensen, Elizabeth Y. Rula, Pina C. Sanelli

**Affiliations:** ^1^ Siemens Medical Solutions USA Inc. Malvern Pennsylvania USA; ^2^ Northwell New York New York USA; ^3^ Department of Radiology Donald and Barbara Zucker School of Medicine at Hofstra/Northwell Hempstead New York USA; ^4^ Department of Radiology Albert Einstein College of Medicine, Montefiore Health System New York New York USA; ^5^ Department of Radiology Boston Medical Center and Boston University Chobanian & Avedisian School of Medicine Boston Massachusetts USA; ^6^ Department of Radiology and Imaging Sciences Emory University School of Medicine Atlanta Georgia USA; ^7^ Department of Radiology Ochsner Health System New Orleans Louisiana USA; ^8^ Division of Gastroenterology and Hepatology Mayo Clinic Rochester Minnesota USA; ^9^ Harvey L. Neiman Health Policy Institute Reston Virginia USA; ^10^ Institute of Health System Science, The Feinstein Institutes for Medical Research Manhasset New York USA

**Keywords:** colorectal cancer, cost‐effectiveness analysis, CT colonography, racial disparity, screening adherence

## Abstract

**Background:**

Given the Centers for Medicare and Medicaid Services' coverage of screening CT colonography (CTC) beginning in January 2025, we evaluated the cost‐effectiveness of CTC for colorectal cancer (CRC) screening by race (Black and White) and gender, considering real‐world screening adherence.

**Methods:**

A microsimulation model compared CRC screening strategies in average‐risk adults by race and gender, incorporating 2010–2019 U.S. data on disease progression and real‐world screening adherence for colonoscopy and fecal immunochemical test (FIT). Five strategies were compared: (1) status quo (choice between colonoscopy and FIT); (2) CTC every 5 years; (3) colonoscopy every 10 years; (4) annual FIT; and (5) multitarget stool DNA test every 3 years, plus no screening. Lifetime costs, quality‐adjusted life years gained (QALYG), and incremental cost‐effectiveness ratios were projected. A willingness‐to‐pay threshold of $100,000/QALYG was used.

**Results:**

Under the status quo, Black adults showed higher CRC cases and greater utilization for FIT over colonoscopy than White adults. Compared to the status quo, the CTC strategy yielded more QALYG and fewer CRC cases among Black adults, but fewer QALYG and more CRC cases among White adults. Both status quo and CTC strategies outperformed other strategies across races. The CTC strategy was the dominant strategy for Black adults. For White adults, the status quo was cost‐effective with incremental cost‐effectiveness ratios of $34,998–$73,428/QALYG, while the CTC strategy was cost saving compared to no screening.

**Conclusions:**

CTC could be cost‐effective for CRC screening in Black adults under real‐world screening adherence, supporting Medicare coverage to address specific population needs and structural barriers to screening.

## 1 Introduction

Colorectal cancer (CRC) is one of the most common cancers in the U.S. [1, 2] Its medical costs are projected to be $26 billion by 2025, ranking second highest among all cancers [3]. Screening can prevent CRC by identifying and removing precursor adenomatous polyps [4, 5]. The U.S. Preventive Services Task Force (USPSTF) recommends CRC screening, including visualization tests (colonoscopy, CT colonography [CTC], flexible sigmoidoscopy) and stool‐based tests (fecal immunochemical tests [FIT], high‐sensitivity guaiac fecal occult blood tests, multitarget stool DNA test [MT‐sDNA]) [4]. Overall, colonoscopy accounts for > 80% and stool‐based tests for > 10% of screenings, with colonoscopy considered the reference standard for its superior detection of adenomas, sessile serrated lesions, and cancers [4, 6].

Despite an overall decline in CRC incidence, non‐Hispanic Black (Black) adults continue to experience higher incidence (42/100,000 population) than non‐Hispanic White (White) adults (36/100,000 population) [1]. Screening access, utilization, and preference likely play a key role in the difference [7]. Black adults more frequently choose stool‐based tests, particularly FIT, while White adults more often choose colonoscopy for CRC screening [8, 9, 10]. This disparity may be due to structural barriers—such as lack of insurance coverage, limited physician recommendations, or infrequent primary care provider visits—that limit access to colonoscopy among Black populations [9, 11, 12]. In contrast, FIT offers advantages including lower cost, reduced time commitment, and lack of need for bowel preparation [9, 11, 12], despite its lower test sensitivity for precancerous polyps and cancer than visualization tests [13, 14, 15].

CTC is a promising alternative screening test, offering better adenoma detection than stool‐based tests and colonoscopy‐equivalent cancer detection with reduced screening burden [13, 15, 16]. Black adults show greater willingness to undergo CTC than White adults, highlighting its potential to address racial disparities while maintaining high‐quality detection [17, 18]. However, screening CTC remains underutilized, partly due to the lack of the Centers for Medicare and Medicaid Services (CMS) coverage through 2024 [19], despite randomized control trials (RCTs) demonstrating improved screening uptake compared to colonoscopy [20, 21].

Prior cost‐effectiveness and efficiency analyses have consistently found CTC less efficient than FIT and colonoscopy for CRC screening in the average‐risk population, though these studies have important limitations [22, 23, 24]. Most studies focused on population‐level analysis with perfect or hypothetical imperfect screening adherence rates without considering demographic differences [14, 22, 23, 24, 25]. In recent years, the Cancer Intervention and Surveillance Modeling Network (CISNET) CRC Working Group began incorporating biological differences through natural disease progression in stratified analyses by race (Black and White) and gender [14, 26, 27]. However, these CISNET studies maintained uniform adherence assumptions across demographic groups, overlooking behavioral variations that might impact real‐world screening effectiveness [14, 27]. One study did account for real‐world adherence but only at the population level without demographic stratification [28].

Given that screening adherence varies by race and gender, and these differences directly impact the comparative effectiveness of screening modalities [7, 14, 26], demographic‐stratified analyses are warranted for developing equitable screening policies. In the literature, the differences in screening adherence between Black and White populations are well documented [7, 26]. As such, in this study, we aimed to assess the cost‐effectiveness of CTC under real‐world screening adherence, stratified by race (Black and White) and gender, compared to the status quo practices and other USPSTF‐recommended screening strategies.

## 2 Methods

### 2.1 Overview

We developed a microsimulation model to evaluate the cost‐effectiveness of CRC screening strategies in average‐risk U.S. adults by race and gender. The model simulated annual disease progression and screening adherence for a cohort of 40‐year‐olds over their lifetimes [14, 26], calibrated to 2010–2019 data from the Surveillance, Epidemiology, and End Results (SEER) Program and National Health Interview Survey (NHIS) [2, 29]. Following the 2021 USPSTF guidelines [4], we evaluated five screening strategies, including the status quo and four single‐modality strategies. The status quo strategy referred to the current real‐world utilization of colonoscopy and FIT for CRC screening. The single‐modality strategies in which only a selected screening modality was offered to the population included CTC‐only (CTC every 5 years), colonoscopy‐only (colonoscopy every 10 years), FIT‐only (annual FIT), and MT‐sDNA‐only (MT‐sDNA every 3 years). No screening was simulated for comparison [14, 26, 30].

From a limited societal perspective [31], we projected lifetime costs and quality‐adjusted life years gained (QALYG) relative to no screening as the primary outcomes, both discounted at 3% annually [32]. Secondary outcomes included CRC incidence, screening test utilization, and total number of colonoscopies (i.e., screening, diagnostic, and surveillance) performed. Model parameters for diagnostic accuracy, complications, costs, and utilities were derived from the literature (Tables 1 and S1) [14, 15, 20, 24, 33, 34, 35, 36, 37, 38, 39, 40, 41, 42, 43]. We developed the simulation model in Python 3.11.8 and conducted cost‐effectiveness analysis (CEA) using the dampack package in R [44]. The institutional review board was not required because no individual data were used.

**TABLE 1:** Base case, range, distribution, and sources of parameters.

Parameter	Base case (range)	Distribution	References
*Test characteristics (patient‐level)*			
Colonoscopy			
Sensitivity			
Diminutive adenoma (1–5 mm)	75% (70%–79%; 100%)	Beta (229, 76)	[14, 33]
Small adenoma (6–9 mm)	85% (80%–92%; 100%)	Beta (187, 33)	[14, 33]
Large adenoma (≥ 10 mm)	95% (93.1%–99.5%; 100%)	Beta (559, 29)	[14, 33]
CRC	95% (93.1%–99.5%; 100%)	Beta (559, 29)	[14]
Specificity	86%^a^	—	[14]
CTC			
Sensitivity			
Diminutive adenoma (1–5 mm)	0% (0%–48%; 50%)^b^	Beta (0.03, 3.01)	[34, 35]
Small adenoma (6–9 mm)	78.6% (66.1%–87.3%; 100%)	Beta (38, 10)	[34]
Large adenoma (≥ 10 mm)	87.9% (82.1%–92.0%; 100%)	Beta (126, 17)	[34]
CRC	96.1% (82.1%–100%; 100%)	Beta (126, 17)	[15]; expert opinion
Specificity	88%^c^	—	[15]
FIT			
Sensitivity			
Diminutive adenoma (1–5 mm)	7.6% (6.7%–8.6%; 20%)	Beta (218, 2645)	[36]
Small adenoma (6–9 mm)	7.6% (6.7%–8.6%; 20%)	Beta (218, 2645)	[36]
Large adenoma (≥ 10 mm)	23.8% (20.8%–27.0%; 100%)	Beta (169, 541)	[36]
CRC	73.8% (62.3%–83.3%; 100%)	Beta (46, 16)	[36]
Specificity	96.4%	—	[36]
MT‐sDNA			
Sensitivity			
Diminutive adenoma (1–5 mm)	17.2% (15.9%–18.6%; 30%)	Beta (495, 2385)	[36]
Small adenoma (6–9 mm)	17.2% (15.9%–18.6%; 30%)	Beta (495, 2385)	[36]
Large adenoma (≥ 10 mm)	42.4% (38.7%–46.2%; 100%)	Beta (278, 378)	[36]
CRC	92.3% (84%–97%; 100%)	Beta (51, 4)	[36]
Specificity	89.8%	—	[36]
*Screening behavior*			
Screening initiation			
Relative risk of initiating screening colonoscopy compared to FIT	0.29 (0.25–0.34)	Beta (112.99, 276.62)	[20]
Relative risk of initiating FIT compared to screening colonoscopy	3.45 (2.94–4.00)	PERT (mode = 3.45, min = 2.94, max = 4)	[20]
Relative risk of initiating CTC compared to screening colonoscopy	1.80 (1.46–2.35)	PERT (mode = 1.8, min = 1.46, max = 2.35)	[20]
Relative risk of initiating CTC compared to FIT	0.53 (0.46–0.61)	Beta (89.64, 79.49)	[20]
Follow‐up adherence for diagnostic colonoscopy			
CTC	97.7% (80%–100%)	Beta (7.46, 0.18)	[20]
FIT	48.7% (20%–100%)	Beta (2076.84, 2187.72)	[37]
MT‐sDNA	66.6% (50%–100%)	Beta (2528.77, 1268.18)	[37]
Adherence to surveillance colonoscopy among patients with adenoma history			
Low risk (history of small adenoma)	44.7% (20%–70%)	Beta (8.81, 10.76)	[38]
High risk (history of large adenoma)	54.6% (40%–70%)	Beta (30.39, 24.87)	[38]

Abbreviations: CRC = colorectal cancer; CTC = CT colonography; FIT = fecal immunochemical test; MT‐sDNA = multitarget stool DNA test.

^a^ The specificity for colonoscopy was assumed imperfect to allow for the detection of nonadenomatous lesions or human errors.

^b^ There is no sensitivity for detection of diminutive adenomas because follow‐up colonoscopy referral is not recommended for detection of lesions < 6 mm in the CTC practice.

^c^ The specificity for CTC was assumed imperfect to allow for detection of nonadenomatous lesions or human errors.

### 2.2 Model Description

We developed a race‐ and gender‐specific microsimulation model to simulate CRC disease progression and screening adherence using annual cycles. CRC disease progression included the following health states: no lesion, adenomas by size (diminutive: 1–5 mm, small: 6–9 mm, large: ≥ 10 mm), cancer by stage (I–IV) categorized as preclinical or clinical, and death (Figure S1) [14, 27].

The model incorporated adherence to the two most commonly used screening modalities in 2010–2019: colonoscopy (~85%) and FIT (> 10%) for each demographic group [6]. Adherence to screening colonoscopy and FIT was modeled separately (Figure S2) [45]. Each screening modality consisted of three components: (1) initial screening (first‐time use); (2) repeat screening following the guidelines‐recommended interval (colonoscopy every 10 years, FIT annually); and (3) the probability of switching between colonoscopy and FIT at the time of next scheduled screening [46, 47]. The approach distinguishing between initial and repeat screening adherence was adapted from the mammography modeling developed by the CISNET Breast Working Group [46, 47]. This is essential when modeling CRC screening, as individuals have multiple screening options and their initial and repeat screening adherence may differ between modalities. All screening ended after age 75 [48]. Details are provided in Supporting Methods.

### 2.3 Model Calibration Using 2010–2019 Data and Model Validation

We calibrated parameters for disease progression and screening adherence by race and gender, aligning the model outcomes with real‐world trends observed in 2010–2019 SEER and NHIS—a period with well‐established screening practices [2, 29]. This approach differed from CISNET models [14, 24, 26, 27, 30, 42, 49, 50, 51], which calibrated CRC disease progression alone and were calibrated to historical periods with no (1975–1979) or minimal screening (1990–1994) in the U.S. population [14, 24, 26, 27, 30, 42, 49, 50, 51, 52].

Calibration targets included both disease outcomes and screening utilizations. Disease‐related targets included age‐specific CRC incidence by race and gender, race‐ and gender‐specific stage distribution by screening (2011–2015) and non‐screening (1975–1979) eras, age‐ and gender‐specific adenoma prevalence, and adenoma detection by size. Screening utilization targets included the proportion of individuals who had ever undergone colonoscopy or stool‐based testing, those who had both, and recency of the last test, all stratified by race, gender, and age. Calibration details and calibrated parameters are provided in Supporting Methods and Tables S2, S3.

Tree‐structured Parzen estimator (TPE) sampler was employed in the calibration process [53, 54]. The calibration process was performed separately for each demographic group, and the 100 top‐performing parameter sets, determined by the mean square error, were selected in our analysis. Cross‐model validation was performed by comparing key simulation outcomes to those reported from the CISNET models (Supporting Methods).

### 2.4 Screening Strategy Following 2021 USPSTF Guidelines and Adherence Assumptions

We modified our calibrated screening adherence parameters to align with the 2021 USPSTF recommendation to begin screening at age 45 instead of 50. Thus, for the status quo strategy, we shifted all calibrated adherence parameters five years earlier, starting at age 45. Both initial and repeat screening adherence in the status quo strategy is shown in Table 2.

**TABLE 2:** Annual probabilities of initial and repeat screening adherence by race and gender.

Strategy	Screening modality that individuals would have adopted in the status‐quo strategy							
	White men		Black men		White women		Black women	
	Colonoscopy	FIT	Colonoscopy	FIT	Colonoscopy	FIT	Colonoscopy	FIT
*Status quo strategy*								
Percent of population who already had screening before age 45^a^	22.4%	8.7%	24.3%	9.0%	23.9%	9.1%	20.2%	10.3%
Annual probability of initial screening^b^								
Age 45–49 years	8.1%	3.1%	6.3%	2.6%	8.1%	3.5%	7.0%	2.0%
Age 50–54 years	4.2%	1.6%	3.2%	1.2%	3.9%	1.6%	3.9%	0.9%
Age 55–59 years	2.4%	0.8%	2.1%	0.6%	1.7%	0.7%	2.0%	0.4%
Age 60–64 years	1.3%	0.4%	1.2%	0.4%	0.8%	0.4%	1.1%	0.2%
Age 65–69 years	0.7%	0.2%	0.7%	0.2%	0.4%	0.2%	0.6%	0.1%
Age 70–75 years	0.7%	0.2%	0.7%	0.2%	0.4%	0.2%	0.6%	0.1%
Annual probability of repeat screening^c^								
Age < 65 years	23.9%	28.0%	20.2%	28.4%	30.4%	32.2%	26.0%	33.9%
Age ≥ 65 years	56.5%	56.1%	64.7%	58.7%	56.6%	61.6%	58.7%	64.1%
*CTC every 5 years for all*								
Percent of population who already had screening before age 45^a^	22.4%	8.7%	24.3%	9.0%	23.9%	9.1%	20.2%	10.3%
Initial screening multiplier^d^	1.80	0.53	1.80	0.53	1.80	0.53	1.80	0.53
Annual probability of initial screening^e^								
Age 45–49 years	14.5%	1.7%	11.3%	1.4%	14.6%	1.8%	12.6%	1.0%
Age 50–54 years	7.6%	0.8%	5.8%	0.6%	7.1%	0.9%	7.0%	0.5%
Age 55–59 years	4.4%	0.4%	3.7%	0.3%	3.0%	0.4%	3.6%	0.2%
Age 60–64 years	2.4%	0.2%	2.2%	0.2%	1.5%	0.2%	2.0%	0.1%
Age 65–69 years	1.3%	0.1%	1.3%	0.1%	0.7%	0.1%	1.1%	0.1%
Age 70–75 years	1.3%	0.1%	1.3%	0.1%	0.7%	0.1%	1.1%	0.1%
Odds ratio adjusting for repeat screening CTC for Black adults^f^	—		1.83		—		1.83	
Annual probability of repeat screening								
Age < 65 years	23.9%		31.7%		30.4%		39.2%	
Age ≥ 65 years	56.5%		77.1%		56.6%		72.2%	
*Colonoscopy every 10 years for all*								
Percent of population who already had screening before age 45^a^	22.4%	8.7%	24.3%	9.0%	23.9%	9.1%	20.2%	10.3%
Initial screening multiplier^d^	1.00	0.29	1.00	0.29	1.00	0.29	1.00	0.29
Annual probability of initial screening^e^								
Age 45–49 years	8.1%	0.9%	6.3%	0.7%	8.1%	1.0%	7.0%	0.6%
Age 50–54 years	4.2%	0.5%	3.2%	0.4%	3.9%	0.5%	3.9%	0.3%
Age 55–59 years	2.4%	0.2%	2.1%	0.2%	1.7%	0.2%	2.0%	0.1%
Age 60–64 years	1.3%	0.1%	1.2%	0.1%	0.8%	0.1%	1.1%	0.1%
Age 65–69 years	0.7%	0.1%	0.7%	0.1%	0.4%	0.1%	0.6%	0.0%
Age 70–75 years	0.7%	0.1%	0.7%	0.1%	0.4%	0.1%	0.6%	0.0%
Annual probability of repeat screening								
Age < 65 years	23.9%	23.9%	20.2%	20.2%	30.4%	30.4%	26.0%	26.0%
Age ≥ 65 years	56.5%	56.5%	64.7%	64.7%	56.6%	56.6%	58.7%	58.7%
*Annual FIT for all*								
Percent of population who already had screening before age 45^a^	22.4%	8.7%	24.3%	9.0%	23.9%	9.1%	20.2%	10.3%
Initial screening multiplier^d^	3.45	1.00	3.45	1.00	3.45	1.00	3.45	1.00
Annual probability of initial screening^e^								
Age 45–49 years	27.8%	3.1%	21.7%	2.6%	28.0%	3.5%	24.2%	2.0%
Age 50–54 years	14.6%	1.6%	11.1%	1.2%	13.6%	1.6%	13.4%	0.9%
Age 55–59 years	8.4%	0.8%	7.2%	0.6%	5.7%	0.7%	6.9%	0.4%
Age 60–64 years	4.5%	0.4%	4.3%	0.4%	2.8%	0.4%	3.9%	0.2%
Age 65–69 years	2.5%	0.2%	2.5%	0.2%	1.4%	0.2%	2.1%	0.1%
Age 70–75 years	2.5%	0.2%	2.5%	0.2%	1.4%	0.2%	2.1%	0.1%
Annual probability of repeat screening								
Age < 65 years	28.0%	28.0%	28.4%	28.4%	32.2%	32.2%	33.9%	33.9%
Age ≥ 65 years	56.1%	56.1%	58.7%	58.7%	61.6%	61.6%	64.1%	64.1%
*MT‐sDNA every 3 years for all*								
Percent of population who already had screening before age 45^a^	22.4%	8.7%	24.3%	9.0%	23.9%	9.1%	20.2%	10.3%
Initial screening multiplier^d^	3.45	1.00	3.45	1.00	3.45	1.00	3.45	1.00
Annual probability of initial screening^e^								
Age 45–49 years	27.8%	3.1%	21.7%	2.6%	28.0%	3.5%	24.2%	2.0%
Age 50–54 years	14.6%	1.6%	11.1%	1.2%	13.6%	1.6%	13.4%	0.9%
Age 55–59 years	8.4%	0.8%	7.2%	0.6%	5.7%	0.7%	6.9%	0.4%
Age 60–64 years	4.5%	0.4%	4.3%	0.4%	2.8%	0.4%	3.9%	0.2%
Age 65–69 years	2.5%	0.2%	2.5%	0.2%	1.4%	0.2%	2.1%	0.1%
Age 70–75 years	2.5%	0.2%	2.5%	0.2%	1.4%	0.2%	2.1%	0.1%
Annual probability of repeat screening								
Age < 65 years	28.0%	28.0%	28.4%	28.4%	32.2%	32.2%	33.9%	33.9%
Age ≥ 65 years	56.1%	56.1%	58.7%	58.7%	61.6%	61.6%	64.1%	64.1%

^a^ Estimated from the NHIS 2010–2019.

^b^ Calibrated to match the screening behavior observed in the NHIS 2010–2019.

^c^ Calibrated to match the screening behavior observed in the NHIS 2010–2019.

^d^ Estimates were reported in Sali et al. [20].

^e^ Calculated the annual probability that someone would begin screening at a particular age by taking the corresponding probability from the status‐quo strategy and multiplying it by the relative risk multiplier. The relative risk factor differed between individuals who would have chosen colonoscopy or FIT in the status quo strategy.

^f^ Odds ratio used to adjust the annual probability of repeat screening for the Black population was derived from Hong et al. 2021. We converted the repeat adherence probability to odds and multiplied these odds by the odds ratio. We then converted these adjusted odds back to probabilities.

For single‐modality strategy (CTC‐only, colonoscopy‐only, FIT‐only, MT‐sDNA‐only), we adjusted initial and repeat adherence rates separately, based on individuals' original screening adherence rates under the status quo strategy.

Initial screening adherence rates were derived by applying modality‐specific multipliers to status‐quo values, stratified by individuals' original modality choice. Multipliers were informed by an RCT comparing adherence rates across colonoscopy, FIT, and CTC among individuals who had never participated in a FIT screening program (Table 2) [20]. If only colonoscopy was offered, individuals who originally initiated with colonoscopy retained their status‐quo initial adherence rates, while those who originally initiated with FIT had their adherence rates reduced by a multiplier of 0.29 [20]. If only FIT was offered, individuals who originally initiated with FIT retained their status‐quo adherence rates, while those who originally initiated with colonoscopy had their adherence rates increased by a multiplier of 3.45 [20]. If MT‐sDNA was the only modality available, initial adherence rates for all individuals were assumed to match those of FIT in the status quo, given that both are stool‐based tests with comparable test burden [4]. If CTC was the only modality available, initial adherence rates were adjusted by a multiplier of 1.8 for individuals who originally started with colonoscopy and 0.53 for those who originally started with FIT [20].

Repeat adherence rates were also derived from modality‐specific repeat screening parameters from calibration (Table 2). Colonoscopy and FIT strategies used their respective repeat adherence rates from the status quo. The MT‐sDNA strategy was assumed to have the same repeat adherence as FIT. For the CTC strategy, we assumed repeat adherence rates among White adults were the same as their repeat colonoscopy adherence rates in the status quo. For Black adults, their adherence rates were increased by applying an odds ratio of 1.83 to their repeat colonoscopy adherence rates in the status quo, based on evidence of higher CTC use among Black adults than White adults [18].

Pathways of diagnostic colonoscopy and surveillance colonoscopy for those with prior adenoma history were considered in the model (Supporting Methods). Diagnostic follow‐up adherence differed by modality (CTC: 97.7%; FIT: 48.7%; MT‐sDNA: 66.6%) [20, 37] but not by demographic groups because our study focused on racial differences in screening adherence.

### 2.5 Costs and Utilities

We derived costs from the literature and 2024 Medicare fee schedules (Supporting Methods and Tables S4, S5). Costs for screening procedures requiring healthcare facility visits (colonoscopy and CTC) encompassed medical procedures, bowel preparation, and time costs for patients and escorts [24, 42, 55, 56, 57, 58]. Stool‐based tests included test costs plus one hour of patient time [24, 42, 59]. Colonoscopy‐related complications and cancer care costs (e.g., medical costs and time costs) were also accounted [24, 42]. All costs were adjusted to 2024 U.S. dollars using the medical care component of the consumer price index [31]. Literature‐based disutilities were applied to screening, surveillance, diagnostic, complications, treatment, and cancer care (Supporting Methods and Table S6) [24, 42].

### 2.6 Cost‐Effectiveness Analysis and Simulation

We conducted CEAs by race and gender under real‐world adherence [31]. Strategies were ranked by total costs in ascending order, followed by eliminating dominated strategies—those generating fewer QALYG than less costly alternatives. For the remaining undominated strategies, we calculated the incremental cost‐effectiveness ratios (ICERs) of a strategy as the ratio of incremental costs to incremental QALYG relative to the previous less costly strategy. Using a willingness‐to‐pay (WTP) threshold of $100,000/QALYG [60, 61], the cost‐effective strategy had the highest ICER below this threshold. Supplementary CEAs assuming perfect adherence were also conducted.

We conducted simulations for each strategy using a cohort of 500,000 adults over their lifetimes. Each simulation incorporated the top 100 calibrated parameter sets specific to each demographic group.

### 2.7 Sensitivity Analyses

One‐way sensitivity analyses tested the robustness of CEA results by varying individual parameters. Strategies were compared using the net monetary benefit, calculated as WTP×QALYGs−Costs, was reported for each strategy [31]. These analyses focused on strategy‐specific screening adherence, test performance, and costs, using the wider parameter ranges specified in Tables 1 and S1. Probabilistic sensitivity analysis (PSA) was conducted using 5000 parameter sets sampled from realistic uncertainty distributions, which were derived from the narrower ranges in Tables 1 and S1. For each PSA sample, we ran 100 simulations using the top‐performing calibrated parameter sets to account for calibration uncertainty. Costs and QALYs for each PSA set were averaged across the 100 simulations. We estimated the proportion of simulations in which each strategy was cost‐effective across WTP thresholds from $10,000 to $500,000/QALYG.

## 3 Results

### 3.1 Simulation Outcomes

The calibrated baseline model fits and model validation are reported in Tables S7, S8 and Figures S3–S6. Without screening, CRC cases/1000 adults were 84 in White men, 67 in Black men, 82 in White women, and 78 in Black women (Table 3).

**TABLE 3:** Cost‐effectiveness analysis under real‐world screening, diagnostic, and surveillance adherence by race and gender.

Strategies	Number of CRC cases per 1000 population	Number of all screening tests per 1000 population	Number of non‐colonoscopy screening tests per 1000 population	Number of screening colonoscopies per 1000 population	Number of all colonoscopies per 1000 population	Initial screening uptake by age 75 (%)	QALYG per 1000 population	Cost per 1000 population (million $)	Incremental cost‐effectiveness ratio
*White men*									
No screening	84	—	—	—	—	—	0	$8.10	—
Screening strategies									
CTC‐only	36	1748	1748	0	555	88%	64	$5.13	
Status quo	31	2625	1514	1111	1380	79%	68	$5.41	$73,428
Colonoscopy‐only	41	885	0	885	1085	73%	55	$5.77	Dominated
FIT‐only	56	8007	8007	0	365	98%	42	$6.08	Dominated
MT‐sDNA‐only	55	2993	2993	0	423	98%	39	$6.90	Dominated
*Black men*									
No screening	67	—	—	—	—	—	0	$6.86	—
Screening strategies									
CTC‐only	31	1681	1681	0	500	81%	58	$4.78	Dominant
Status quo	33	2290	1393	897	1117	72%	54	$5.31	Dominated
FIT‐only	47	7592	7592	0	333	94%	39	$5.44	Dominated
Colonoscopy‐only	40	691	0	691	846	67%	42	$5.53	Dominated
MT‐sDNA‐only	45	2955	2955	0	399	94%	38	$6.15	Dominated
*White women*									
No screening	82	—	—	—	—	—	0	$7.21	—
Screening strategies									
CTC‐only	37	1967	1967	0	523	85%	53	$4.90	
Status quo	30	2998	1784	1214	1445	77%	58	$5.08	$34,998
FIT‐only	49	11,432	11,432	0	419	97%	41	$5.26	Dominated
Colonoscopy‐only	40	923	0	923	1088	70%	46	$5.35	Dominated
MT‐sDNA‐only	49	4063	4063	0	484	97%	37	$6.31	Dominated
*Black women*									
No screening	78	—	—	—	—	—	0	$7.49	—
Screening strategies									
CTC‐only	33	2087	2087	0	534	83%	68	$4.95	Dominant
Status quo	33	2790	1716	1075	1290	71%	65	$5.40	Dominated
FIT‐only	48	10,500	10,500	0	392	95%	50	$5.44	Dominated
Colonoscopy‐only	42	832	0	832	985	67%	52	$5.66	Dominated
MT‐sDNA‐only	47	3854	3854	0	462	95%	47	$6.45	Dominated

Abbreviations: CRC = colorectal cancer; CTC = CT colonography; FIT = fecal immunochemical test; MT‐sDNA = multitarget stool DNA test; QALYG = quality‐adjusted life years gained relative to no screening.

The status quo strategy reduced CRC incidence across all demographic groups (White men: 31 cases/1000 adults; Black men: 33 cases/1000 adults, White women: 30 cases/1000 adults; Black women: 33 cases/1000 adults). The status quo strategy generated the highest QALYG/1000 adults in White men (68), followed by Black women (65), White women (58), and Black men (54). Screening utilization (i.e., tests/1000 adults) was higher among women (White: 2998; Black: 2790) than men (White: 2625; Black: 2290). White adults had more screening colonoscopies/1000 adults (men: 1111; women: 1214) than Black adults (men: 897; women: 1075). FIT‐to‐colonoscopy ratios were 1.55–1.60 for Black adults compared to 1.36–1.47 for White adults.

Compared to the status quo strategy, the CTC‐only strategy had varying effectiveness across demographic groups. Among White adults, the CTC‐only strategy yielded more CRC cases/1000 adults (36–37) and fewer QALYG/1000 adults (men: 64; women: 53) than the status quo strategy. Among Black adults, the CTC‐only strategy produced similar or fewer CRC cases/1000 adults (31–33) and more QALYG/1000 adults (men: 58; women: 68) than the status quo. CTC utilization (i.e., CTC tests/1000 adults) was 1.6 times higher than the screening colonoscopy utilization in the status quo strategy among White adults and nearly double (1.9 times) among Black adults.

All screening strategies yielded more QALYG at lower costs than no screening, but the colonoscopy‐, FIT‐, and MT‐sDNA‐only strategies were less effective than both the status quo and CTC strategies across all demographic groups. Strategy costs varied substantially: the CTC‐only strategy was the least costly, followed by the status quo strategy, and the MT‐sDNA‐only strategy was the most costly. For colonoscopy utilization (i.e., all colonoscopies/1000 adults), the status quo strategy showed a higher utilization than the colonoscopy‐only strategy due to its higher overall screening uptake and the flexibility for individuals to switch between screening modalities at each screening interval. Comparing non‐colonoscopy strategies, colonoscopy utilization was the highest for the CTC‐only strategy, followed by the MT‐sDNA‐ and FIT‐only strategies.

### 3.2 CEA

Among White adults under real‐world adherence, only the status quo and CTC‐only strategies were undominated, with the status quo strategy being cost‐effective compared to the CTC‐only strategy (ICERs = $73,428 and $34,998 per QALYG in White men and women, respectively). In this population, the CTC‐only strategy yielded more QALYs at a lower cost (i.e., cost saving) than no screening. Among Black men and women under real‐world adherence, the CTC‐only strategy was the dominant strategy among all strategies.

Perfect adherence yielded consistent findings across demographic groups: FIT‐only was cost‐effective compared to the colonoscopy‐only strategy, which exceeded the WTP threshold (ICERs: $234,659–$318,444/QALYG across demographic groups; Table S9). Both the CTC‐ and MT‐sDNA‐only strategies were dominated.

### 3.3 Sensitivity Analyses

**FIGURE 1:**
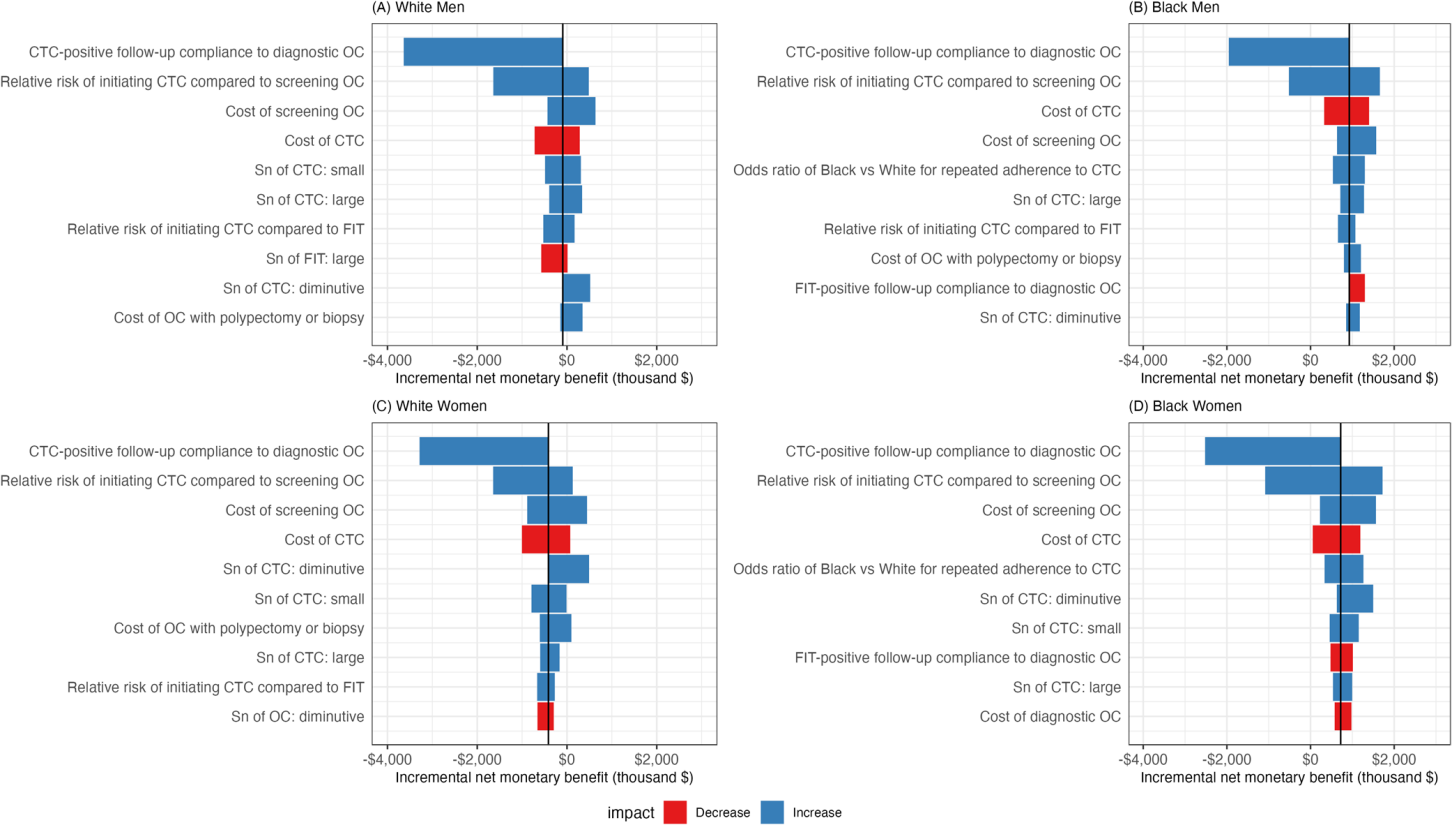
One‐way sensitivity analysis: The top 10 most influential parameters comparing between the CT colonography‐only (CTC every 5 years) and status quo strategy by White men (A), Black men (B), White women (C), and Black women (D). Vertical line represents the incremental net monetary benefit (NMB) of CT colonography (CTC)‐only relative to the status quo strategy using base case values. Positive incremental NMB favored CTC‐only; negative values favored the status quo strategy. Blue horizontal bars imply that parameters are positively associated with incremental NMB; red horizontal bars suggest that parameters are negatively associated with incremental NMB.

One‐way sensitivity analysis compared the status quo and CTC‐only strategies using incremental net monetary benefit because these two strategies consistently outperformed others under real‐world adherence (Figure 1). Positive incremental net monetary benefit favored the CTC‐only strategy; negative values favored the status quo strategy. Varying CTC‐positive diagnostic follow‐up adherence did not change the optimal strategy for White adults, while decreasing adherence shifted the optimal strategy from the CTC‐only to the status quo strategy for Black adults. Consistent across groups, a higher multiplier of initial CTC screening increasingly favored the CTC‐only strategy over the status quo strategy. For costs, higher colonoscopy costs favor the CTC‐only strategy, and higher CTC costs favor the status quo strategy, consistent across groups. Among Black adults, while the odds ratio of repeat screening CTC adherence emerged as the fifth most influential parameter, the CTC‐only strategy was favored over the parameter range.

**FIGURE 2:**
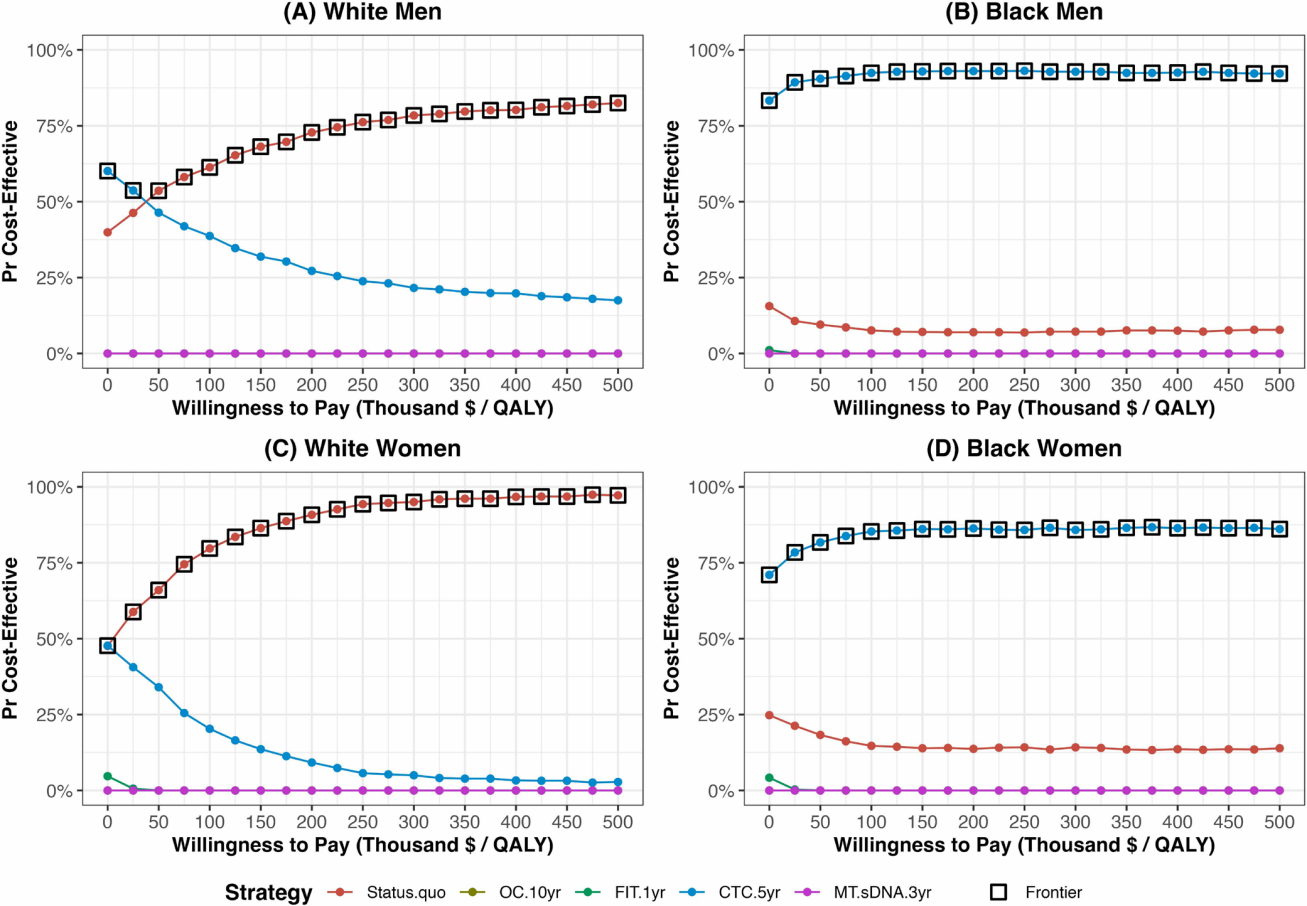
Probabilistic sensitivity analysis by race and gender. The frontier indicates the most likely cost‐effective strategy at each willingness‐to‐pay threshold. For White men (A), the most likely cost‐effective strategy switched from the CT colonography (CTC)‐only to the status quo strategy around the threshold of $30,000/QALYG. For Black men (B), the CTC‐only strategy was the most favorable strategy across all thresholds. For White women (C), the status quo strategy was most likely cost‐effective across all thresholds. For Black women (D), the CTC‐only strategy was the most favorable strategy across all thresholds.

PSA results varied across demographic groups (Figure 2). For White men, the CTC‐only strategy was preferred at WTP thresholds < $30,000/QALYG, beyond which the status quo strategy became more likely to be cost‐effective. Among White women, the status quo strategy was cost‐effective in over 50% of the iterations across all WTP thresholds. In contrast, the CTC strategy maintained robust cost‐effectiveness among Black adults across all WTP thresholds, favored in over 80% and 75% of the iterations for men and women, respectively.

## 4 Discussion

Analysis of real‐world screening adherence shows the CTC‐only strategy emerged as the dominant strategy for Black adults, while both the CTC‐only and status quo strategies could be optimal for White adults depending on resource constraints. These racial differences stemmed primarily from disparities in screening adherence in the status quo strategy. Consistent with CISNET models [26, 42], our model showed that Black adults had lower CRC incidence than White adults in the absence of screening. However, this pattern reversed under the status quo strategy, reflecting recent trends [1]. Screening modality‐specific utilization in the status quo strategy revealed important racial differences. While Black adults had overall screening utilization comparable to or only slightly lower than White adults, they demonstrated greater reliance on FIT than White adults. While review studies showed that FIT alongside flexible sigmoidoscopy is associated with reduced CRC mortality in an RCT [13, 62], FIT alone with its lower sensitivity for adenomas and CRC and low diagnostic follow‐up adherence [13, 14, 15] contributed to the differential effectiveness of the status quo strategy between Black and White adults.

Our findings suggested that CTC can potentially address the limitations of other screening modalities. Compared to stool‐based tests, CTC provided better adenoma detection and higher diagnostic follow‐up adherence, yielding more diagnostic colonoscopy procedures [15, 20, 34, 37]. Compared to screening colonoscopy, CTC achieved higher screening utilization and adherence, particularly among Black adults, while reducing screening burden (e.g., no need for sedation, less time costs, and fewer adverse events) and costs [9, 16, 17, 18, 63]. These comparative advantages made CTC the only single‐modality strategy that could compete with the status quo strategy in our study. As such, CTC outperformed all other strategies in Black adults and could be cost‐effective in White adults in resource‐limited settings. For instance, CTC could be cost‐effective among White men at the WTP threshold of $50,000/QALYG.

Our results diverged from previous CEAs, which suggested that CTC was less cost‐effective than, or even dominated by, FIT or colonoscopy [22, 23, 24, 26]. This discrepancy was due to differences in the assumptions of screening adherence. Previous analyses either assumed perfect screening adherence or did not consider both race‐ and gender‐specific screening adherence [22, 23, 24, 26]. In fact, our sub‐analysis under perfect adherence was consistent with previous studies [22, 23, 24, 26], showing that FIT was the cost‐effective strategy across all groups and dominated CTC while colonoscopy exceeded the threshold of $100,000/QALYG [64]. Thus, CTC was not historically covered by the CMS [65], nor recommended as a potential screening strategy for the average‐risk population by the American College of Physicians in 2023 [66].

Because of imperfect adherence, the availability and coverage of multiple screening strategies is beneficial, as both individual preferences and structural barriers influence the uptake and effectiveness of CRC screening in real‐world conditions [9, 11, 12]. Therefore, offering alternative screening options like CTC—which balances test performance, reduces screening burden, and improves diagnostic follow‐up adherence—may better address a range of patient needs while also mitigating structural barriers in access to care. Our analysis demonstrates the importance of accounting for these multifaceted contributors to screening behaviors, as screening benefits can only be manifested when patients adhere to recommended guidelines. The 2025 CMS decision to cover screening CTC for all Medicare/Medicaid beneficiaries aligns with this perspective [65]. Prior research found large income disparities in the utilization of screening CTC in Medicare beneficiaries when patients were required to pay out‐of‐pocket [19]. Expanding CMS coverage of screening CTC could help reduce financial barriers and benefit both evaluated racial groups: offering Black adults a potentially more feasible option that may improve adherence, and White adults a comparably effective but lower‐cost alternative to the status quo strategy.

Our study has several limitations. First, although we followed the 2021 USPSTF recommendations for CRC screening strategies, our model relied on pre‐2020 data. Post‐2020 SEER and NHIS data were not used to avoid the influence of the coronavirus 2019 pandemic, which significantly disrupted cancer screening programs [67, 68]. Second, the relative risk multipliers used to adjust modality‐specific initial screening probabilities came from an Italian RCT [20]. While not fully generalizable to the U.S., it is the best available data to compare all screening modalities of interest. We addressed this limitation through sensitivity analyses. Despite not being race‐ or gender‐specific, these multipliers were applied to race‐ and gender‐specific status‐quo initial adherences, allowing us to preserve demographic differences in adherence while incorporating modality‐specific multipliers from the trial. Third, CTC, MT‐sDNA, and sigmoidoscopy were not considered in the status quo strategy due to their limited, evolving, or declining use during the 2010–2019 calibration period. Specifically, CTC represented < 3% of screenings, MT‐sDNA was not FDA‐approved until August 2014, and sigmoidoscopy use was steadily declining [6, 29]. Hence, the strategy—annual FIT alongside flexible sigmoidoscopy every 10 years—was also excluded from the analysis. Fourth, our study only focused on comparing Black and White populations as the differences between these two populations are better documented than the other race/ethnicity groups [7, 26]. However, it is recommended that other race/ethnic groups be carefully examined in future studies. Fifth, our CTC test performance estimates, similar to those used in the CISNET models, were based on pre‐2010 studies and may underestimate its current effectiveness given technological advances [15, 34, 35]. Finally, our cost of CTC ($225), based on the 2024 CMS rate for diagnostic CTC, was lower than the 2025 rate, $350, because the analysis was conducted in 2024. However, the updated cost of CTC did not change the results in the one‐way sensitivity analysis.

## 5 Conclusions

Under real‐world screening adherence, CTC emerges as a cost‐effective CRC screening strategy, yielding health benefits in Black adults while also remaining effective for White adults. By offering a balance between test performance and screening burden, CTC can enhance screening adherence among Black adults and provide an effective alternative for White adults. These findings support the recent CMS coverage decisions for CTC screening.

## Author Contributions

**Szu‐Yu Zoe Kao:** conceptualization (lead), data curation (lead), formal analysis (lead), investigation (lead), methodology (lead), project administration (equal), resources (lead), software (lead), validation (lead), visualization (lead), writing – original draft (lead), writing – review and editing (lead). **Maria X. Sanmartin:** conceptualization (equal), investigation (equal), methodology (equal), writing – original draft (equal), writing – review and editing (equal). **Judy Yee:** conceptualization (equal), investigation (equal), methodology (equal), writing – review and editing (equal). **Kevin J. Chang:** investigation (equal), writing – review and editing (equal). **Courtney A. Moreno:** investigation (equal), writing – review and editing (equal). **Cecelia Brewington:** investigation (equal), writing – review and editing (equal). **David H. Bruining:** investigation (equal), writing – review and editing (equal). **Eric W. Christensen:** conceptualization (equal), investigation (equal), methodology (equal), writing – review and editing (equal). **Elizabeth Y. Rula:** conceptualization (equal), investigation (equal), methodology (equal), writing – review and editing (equal). **Pina C. Sanelli:** conceptualization (equal), investigation (equal), methodology (equal), project administration (equal), supervision (equal), writing – original draft (equal), writing – review and editing (equal).

## Ethics Statement

The institutional review board was not required because all input parameters were derived from publicly available data and no individual data were used.

## Conflicts of Interest

Dr. Kao is employed by and owns stock of Siemens Medical Solutions USA Inc. Dr. Sanmartin received research grants outside the submitted work from the American Heart Association, the Feinstein Institutes for Medical Research, the Northwell Katz Institute for Women's Innovations Grant Program, the American College of Radiology's Harvey L. Neiman Health Policy Institute, Siemens Healthineers, and NIH National Institute of Neurological Disorders and Stroke. Dr. Yee received a research grant from GE Healthcare. Dr. Chang received honoraria from Bracco Diagnostics, Koninklijke Philips N.V., and Medality, and is an investor in Theromics Inc. Dr. Brewington received research support for colon, lung, and breast cancer screening from Bracco. Dr. Bruining received research support from Nextrast and was consulting for Janssen. Dr. Sanelli received research funding outside the scope of the submitted work from the NIH National Institute of Neurological Disorders and Stroke (NINDS) and National Institute of Allergy and Infectious Diseases (NIAID), the American College of Radiology's Harvey L. Neiman Health Policy Institute, American Heart Association, Blue Rock Therapeutics, and Siemens Healthineers. Drs. Moreno, Christensen, and Rula have no conflicts of interest to declare.

## Supporting information


**Data S1:** cam471290‐sup‐0001‐DataS1.docx.

## Data Availability

All model parameters were derived from publicly available data sources and the literature. All data and model outputs are available upon request.
