# Supplementary material for: Cost‐Effectiveness of CT Colonography Under Real‐World Colorectal Cancer Screening Adherence for Black and White Populations
Source: Cancer Med. 2025 Nov 12;14(21):e71290. doi: 10.1002/cam4.71290 (PMC12612557; doi:10.1002/cam4.71290)
Supplement: Supplementary file 1 — Data S1: cam471290‐sup‐0001‐DataS1.docx. [file CAM4-14-e71290-s001.docx]

**Supporting Materials**

**Supporting Methods**

**CRC disease progression in the absence of screening**

The model began at age 40 in a cohort of average-risk U.S. adults and simulated CRC progression through the adenoma–carcinoma sequence. Health states included: no lesion; adenomas by size (diminutive: 1–5 mm, small: 6–9 mm, large: ≥10 mm); preclinical and clinical cancers (stages I–IV); and death (Supporting Fig. 1).

At model entry, individuals were assigned to one of the following initial health states: no lesion, adenoma (by size), or preclinical cancer (stages I–IV).^1–7^ Initial adenoma prevalence at age 40 was 26% for men and 18% for women, with no racial differences due to lack of race-specific data.^8^ Among those with adenomas, 62% were diminutive, 29% small, and 9% large.^9^ A small proportion had undetected preclinical CRC, with prevalence declining by stage, modeled as:

$\begin{aligned} \rho_{i}=\rho_{I}\cdot\kappa^{i-1}\#\left[ 1 \right]\#\# \end{aligned}$

where *i* denotes preclinical stage I–IV and $\kappa\in(0, 1)$ is a stage-based decay factor.

In the absence of screening, individuals could progress to the next more advanced disease states, have cancer detected via symptoms, or die within a given cycle. Disease progression was tracked based on the most advanced lesion present. Individuals without lesions/polyps could develop diminutive adenomas over time. Following Rutter et al.,^9^ we assumed that the annual rate of developing adenomas increased with age, with growth rate changed at ages 40, 60, and 70. The growth rate function was defined as:

$\begin{aligned} f_{1}(t)=\alpha_{0}+\alpha_{40}\cdot1_{\left( 40, 100 \right]}(t)\cdot min(t-40, 60-40)+ \\ \alpha_{60}\cdot1_{\left( 60, 100 \right]}(t)\cdot min(t-60, 70-60)+ \\ \alpha_{70}\cdot1_{\left( 70, 100 \right]}(t)\cdot(t-70)\#\left[ 2 \right]\#\# \end{aligned}$

where $\alpha_{0}$ is the baseline rate at age 40; $\alpha_{40}$, $\alpha_{60}$, $\alpha_{70}$ represent incremental age effects; $1_{\left( a, 100 \right]}(t)$ is an indicator function equal to 1 when age *t > a*. We modified the function’s lower bound to age 40 to align with our model initialization.

Adenomas progressed sequentially from diminutive to small, then large, and ultimately to preclinical cancer stage I, with annual growth rates varying by adenoma size.^1,9,10^ Growth rates were influenced by both age and the duration since adenoma onset.^1,9,10^ The annual growth rate was modeled as:

$\begin{aligned} f_{2}\left( t \right)=\beta_{0}+\beta_{age}\cdot\left( t-40 \right)+\beta_{dur}\cdot T+\beta_{small}\cdot Small+\beta_{large}\cdot Large\#[3] \end{aligned}$

where $\beta_{0}$ is the base rate at age 40 for diminutive adenomas; $\beta_{age}$ captures the effect of aging; $\beta_{dur}$ reflects time since initial adenoma onset (*T*). Coefficients, $\beta_{small}$ and $\beta_{large}$, adjust the rate for small and large adenomas, respectively, with indicator variables *Small* and *Large* representing adenoma size category.

Individuals in a preclinical cancer stage could either progress to a more advanced stage or be incidentally diagnosed due to symptoms, at which point they would enter the corresponding clinical stage.^1–7^ The annual progression rate between preclinical stages was modeled as a function of age and time since entering the current stage,^1,9,10^ specifically:

$\begin{aligned} g_{j}\left( t \right)=\lambda_{j0}+\lambda_{j1}\cdot\left( t-40 \right)+\lambda_{j2}\cdot T_{j} \#[4] \end{aligned}$

, where *j* = 1 (preclinical stage I to II), 2 (preclinical stage II to III), or 3 (preclinical stage III to IV). Parameters, $\lambda_{j0}$, $\lambda_{j1}$, and $\lambda_{j2}$, represent the base rate, age effect, and duration effect, respectively.

The annual incidental detection rate was modeled to reflect increasing symptom presentation with age, cancer stage, and time spent in each preclinical stage.^11,12^ It was defined as:

$$\begin{aligned} h_{j}\left( t \right)=\delta_{0}+\delta_{age}\cdot\left( t-40 \right)+\left( \sum_{j} \delta_{j}\cdot1_{\left( stage\leq j \right)} \right)\cdot T_{j}\#\left[ 5 \right] \end{aligned}$$

where *j*=I–IV corresponds to transitions from preclinical to clinical cancer at the same stage. Parameters $\delta_{0}$ and $\delta_{age}$ denote the base detection rate and age effect, respectively. The term $\delta_{j}$ captures the contribution of each cancer stage, and $1_{\left( stage\leq j \right)}$ indicates whether the individual is at or below preclinical stage *j*. This cumulative component, $\left[ \sum_{j} \delta_{j}\cdot1_{\left( stage\leq j \right)} \right]\cdot T_{j}$, adjusts detection likelihood by both cancer stage and duration in that stage, controlling for age.

Mortality rates were derived from two sources: cancer-specific mortality from individuals diagnosed in 2000-2009 in the SEER data (10-year relative survival rates by stage and age at diagnosis, race, and gender)^11^ and other-cause mortality from the 2019 U.S. life tables (by race and gender).^13^ All individuals could die from other causes, but only individuals in clinical cancer could die from cancer-specific causes.

All annual rates were converted into probability based on exponential distribution assumption $1-\exp\left( -rate \right)$. All parameters in disease progression were estimated via calibration by race and gender.

**Modeling screening adherence in 2010-2019**

Screening adherence incorporated both colonoscopy and fecal immunochemical test (FIT) behaviors. We modeled adherence as a state-transition process^14^ starting at age 50 (Supporting Fig. 2), consistent with the 2016 US Preventive Service Task Force (USPSTF) guidelines.^15^ Each modality consisted of three components: (1) initial screening (first-time use), (2) repeat screening following the guidelines-recommended interval (every 10 years for colonoscopy, annually for FIT), and (3) the probability of switching between colonoscopy and FIT at the time of next scheduled screening.^14,16,17^

Although the recommended starting age was 50, NHIS data showed non-negligible screening utilization before that age.^18^ To account for early screening initiation, we used NHIS data to classify individuals entering the model at age 50 into one of three categories: colonoscopy initiators, FIT initiators, or non-initiators.^14,19,20^ This allowed the model to reflect real-world variation in screening behavior prior to age 50 and to determine future eligibility and timing for rescreening.

Based on NHIS data for adults aged 45-49, colonoscopy uptake was 22% for White men, 24% for Black men, 24% for White women, and 20% for Black women.^18^ For these individuals, we assumed colonoscopy was performed between ages 45-49, with the next screening screening scheduled 10 years later. Similarly, FIT uptake prior to age 50 was 9% for White men, Black men, and White women, and 10% for Black women.^18^ We assumed these individuals underwent FIT at age 49 and were eligible for the next FIT scheduled at age 50.

As individuals aged, we assumed that the probability of initial screening declined. Annual initial screening probabilities for colonoscopy and FIT were modeled by age category: 50-54, 55-59, 60-64, 65-69, 70-75. Initial screening probability with modality *i* (where *i = C* for colonoscopy or *F* for FIT) at age *t* was defined as:

$$\begin{aligned} L_{i}\left( t \right)=\zeta_{i50}\cdot\left( 1_{j=0}+\prod_{j} \kappa_{ij}\cdot1_{[1, j]} \right)\#[6] \end{aligned}$$

where $\zeta_{i50}$ is the base initial screening probability at ages 50-54, and *j* indexes the age group (0 = 50–54, 1 = 55–59, ..., 4 = 70–75). The term $\kappa_{ij}$ denotes a multiplicative adjustment factor (bounded between 0 and 1) that reduces initial probability in later age group. The indicator function $1_{[1, j]}$ determines whether the individual is within each successive age category, allowing the model to capture age-specific declines in initial screening.

Individuals who initiated screening could either continue with their initial modality (colonoscopy every 10 years or annual FIT) based on **repeat screening adherence**, or switch to a different modality at the next screening interval. Repeat screening adherence was assumed to increase after age 65 due to improved access through Medicare. The adherence probability for repeat screenings was defined as:

$$\begin{aligned} R_{i}\left( t \right)=\gamma_{i65}\cdot\left( \tau_{i}\cdot1_{\left[ 50, 65 \right)}+1_{\left[ 65,75 \right]} \right)\#[7] \end{aligned}$$

where $\gamma_{i65}$ is the adherence probability for individuals aged ≥65 for modality *i* (C = colonoscopy, F = FIT), and $\tau_{i}$, between 0 and 1, is a multiplicative factor reducing adherence for those under age 65. The probability of switching between modalities at the next screening interval was assumed constant over time and varied by initial modality. All parameters were estimated separately by race and gender through model calibration.

**Model calibration**

Our model simultaneously calibrated parameters for disease progression and screening adherence by race and gender (Supporting Tables 2-3). Two categories of calibration targets were used:

Disease-related targets:

- Age-specific CRC incidence by race and gender in 2010-2019^11^
- Stage distribution by race and gender in the no screening era (1975-1979)^21^ to inform the stage at diagnosis via symptomatic detection
- Stage distribution by race and gender in the recent screening era in 2011-2015^11^ to inform stage at diagnosis via screening detection
- Prevalence of adenoma by age and gender in no screening era^8^ to inform natural adenoma growth in the absence of screening
- Size distribution of adenoma at screening detection^9^

Screening utilization targets estimated from the 2010-2019 NHIS:^18^

- Age-specific proportion of population who had ever undergone colonoscopy *or* stool-based testing, by race and gender
- Age-specific proportion of population who had undergone both colonoscopy *and* stool-based testing, by race and gender
- Age-specific proportion of population with recent screening following guideline-recommended screening interval: colonoscopy within 10 years or FIT with 1 year

Both lifetime and recent screening utilization targets were used to inform initial and repeat screening adherence, respectively. Because no data source directly reports these components separately, we relied on these two types of targets to indirectly capture different patterns of adherence across demographic groups.

Calibration was conducted separately for each demographic group using the Tree-structured Parzen Estimator (TPE) sampler.^22,23^ Each run began with 300 randomly sampled parameters sets and proceeded with 10,000 model-based iterations of the TPE algorithm. Model performance was assessed using the mean squared error (MSE) between simulated outcomes and calibration targets. We retained the top 100 best fitted parameter sets per group for further analysis.

We performed cross-validation of our model by comparing the key simulation outcomes with those reported from the three CISNET models (SimCRC, CRC-SPIN, MISCAN),^1,24^ including (a) adenoma dwell time, which is the duration between adenoma onset and the time that a patient progress to preclinical cancer stage I; (b) preclinical cancer sojourn time, which is the duration between the onset of preclinical cancer to the time at cancer diagnosis; (c) lifetime CRC incidence per 1,000 population by race and gender.^1,5,25^ For the duration measures (i.e., adenoma dwell time and preclinical cancer sojourn time), only individuals developed adenomas or cancer after the entry of model were accounted in the calculation for cross-validation.

**Screening, diagnostic, and surveillance pathways**

Screening outcomes were determined by literature-derived modality-specific test accuracy (sensitivity and specificity)^1,26–29^ and individual’s most advanced disease status.

For non-colonoscopy screening strategies (CTC, FIT, and MT-sDNA), negative results allowed individuals to remain in their current health states, though disease progression could occur by the end of each cycle. Following positive results, diagnostic colonoscopy was recommended with varying compliance rates: 97.7% for CTC, 66.6% for MT-sDNA, and 48.7% for FIT.^30,31^ In the CTC strategy, only individuals with lesions ≥6 mm or cancer were referred for diagnostic colonoscopy, following current polyp surveillance guidelines.^3,32,33^ Test specificity differed among modalities (CTC: 88%, FIT: 96.4%, MT-sDNA: 89.8%), with the lower specificity of CTC reflecting its ability to detect non-adenomatous lesions, which stool-based tests cannot identify.^3,4^

Unlike non-colonoscopy screening modalities, screening colonoscopy combined detection and intervention in a single procedure, eliminating the need for separate diagnostic colonoscopy after positive findings.^34^ Following screening or diagnostic colonoscopy, management depended on findings. Negative results allowed individuals to remain in their current states. Adenoma detection led to immediate polypectomy and return to no-lesion state, though new adenomas could develop by the end of each cycle.^1,3,7^ Small adenoma detection initiated 5-year surveillance intervals (44.7% adherence), while large adenomas required 3-year intervals (54.6% adherence), both continuing until age 85.^34–37^ Colonoscopy was the modality used in surveillance among those who with adenoma history.^37^ Cancer detection prompted immediate biopsy and transition to clinical cancer stage. Like CTC, colonoscopy has imperfect specificity (86%), leading to some non-adenomatous lesion removal, though these false positives were ultimately classified as normal through histologic examination.^3,4^

We modeled colonoscopy-related complications in two categories: severe gastrointestinal (GI) events and non-severe events.^3,4,38–41^ Severe GI events (perforations, bleeding, or transfusions) could lead to perforation (89.7 per 1,000 events) or perforation-related death (51.9 per 1,000 perforations).^38,40,41^ Non-severe events included GI complications (35%; including paralytic ileus, nausea, abdominal pain) and cardiovascular events (65%), which while non-fatal, increased costs and decreased quality of life.^35,38^ Complication risks varied by procedure type: highest for polypectomy, lowest for screening/diagnostic colonoscopy alone, with biopsy increasing risk by 22% (relative risk of 1.22).^39^ Non-colonoscopy screening modalities carried negligible complication risks.

**Costs of screening, surveillance, and follow-up/diagnostic procedures**

We derived costs associated with screening, surveillance, and diagnostic procedures following the cost estimation method outlined in Peterse et al.^6^ and update the cost of these procedures with the 2024 Medicare fee-for-service payment rates (Supporting Table 4).^42–45^ This method accounted for the cost of medical service, preparation, and patient and escort time by procedure. The total cost estimates and cost components by procedure are provided in Supporting Table 5.

For non-colonoscopy screening procedures, we considered different cost components between stool-based tests (FIT and MT-sDNA) and CTC because the resources and time differed between the two types of tests. Stool-based tests can be conducted at home, whereas CTC requires hospital visit. For stool-based tests, the total cost included the cost of test ($18 for FIT; $509 for MT-sDNA) and the indirect cost of one hour of patient time (median hourly wage $35).^6,43^ For CTC, the total cost comprised the cost of test (approximated based on diagnostic CTC, $225), bowel preparation kit ($110), and the indirect cost of patient time ($400 for 11 hours, excluding 8 hours of sleep).^6,44–46^ The patient time for CTC consisted of the time spent on bowel preparation, travel, waiting or preparation time in hospital, and CTC procedure.^6^

Colonoscopy procedures required hospital visit like CTC but required longer patient time than CTC and assistance during recovery. Thus, the total costs of an colonoscopy procedure included the cost of test, anesthesia, preparation, patient and escort time ^6^. Medicare payment rate was the same, $1053, among screening, diagnostic, and surveillance colonoscopy if polypectomy or biopsy was not performed.^44,45^ With polypectomy or biopsy, the payment rate of a colonoscopy procedure increased to $1447, averaging across 5 HCPCS/CPT codes (45380, 45381, 45382, 45384, 45385) weighted by their total number of claims reported in the cost statistics from the 2024 Medicare hospital outpatient prospective payment system.^44,45^ The cost of anesthesia, higher for diagnostic colonoscopy ($123) than screening and surveillance colonoscopy ($96), included fee for anesthesiologist and the medication, which was parameterized by the price of propofol from the Veteran Affairs Federal Supply Schedule.^42,47,48^ The indirect cost for a colonoscopy procedure was $843 for 24 hours, which included 3.72 hours of escort time and 20.22 hours of patient time excluding 16 hours of sleep.^6^

In general, the total cost was higher for a colonoscopy procedure, varying from $2103 to $2524, than non-colonoscopy screening procedures ($735 for CTC, $53 for FIT, and $544 for MT-sDNA). Indirect cost of patient and escort times were estimated using the 2023 median hourly wage inflated to 2024 US dollars ($35).^46^

**Disutility associated with screening, surveillance, and follow-up/diagnostic procedures**

Disutility, measuring the reduction in quality of life, was accounted for events associated with screening, surveillance and diagnostic procedures were obtained from Peterse et al. and the technical report published by the CISNET Colorectal Cancer Working Group.^1,6^ The sources of utility loss consisted of the loss due to undergoing the test procedure itself, the wait time for test results, and the wait time for diagnostic colonoscopy if non-colonoscopy screening results were abnormal (Supplementary Table 6). Estimates of utility losses were assumed to be additive as events occurred.^1^

Based on the assumptions from the CISNET group, the utility loss resulted from a procedure itself only incurred as patients undergoing a colonoscopy or CTC procedure. Patients experienced utility decrements of 0.12 lasting 36 hours for colonoscopy and 19 hours for CTC, respectively.^1^ We assumed that the utility losses continued during sleep (16 hours for colonoscopy, 8 hours for CTC) while patients prepared for or recovered from the procedure.^1,6^ Waiting for the test results also lead to utility losses, varying with the length of wait time and procedure.^1^ A colonoscopy procedure without polypectomy or biopsy resulted in no additional utility loss attributable to result waiting time because patients received results immediately after the procedure.^1^ However, a colonoscopy procedure with polypectomy or biopsy decreased the utility by 0.033036 for 10 days while patients waited for the results.^1^

For non-colonoscopy screening procedures, while waiting for the results, patients experienced a utility loss of 0.003304 for 3 days for CTC, 7 days for FIT, and 14 days for MT-sDNA.^1^ If the results from non-colonoscopy screening were abnormal, patients waited for 14 days for diagnostic colonoscopy and experience a utility loss of 0.033036 on a daily basis.^1^

Supplementary Tables

Supporting Table 1. Base case, range, distribution, and sources of parameters

| **Parameter** | **Base case (range)** | **Distribution** | **Reference** |  |
| --- | --- | --- | --- | --- |
| **Complications** | | | |  |
| Screening colonoscopy (per 1000 colonoscopies) |  |  |  |  |
| Serious GI events | 2.8 (1.2–4.3) | Beta(16, 5861) | Warren et al., 2009^38^ |  |
| Other events | 19.0 (13.3–24.7) | Beta(47, 2444) | Warren et al., 2009^38^ |  |
| Follow-up/diagnostic colonoscopy (per 1000 colonoscopies) |  |  |  |  |
| Serious GI events | 4.2 (3.3–5.2) | Beta(74, 17498) | Warren et al., 2009^38^ |  |
| Other events | 24.7 (21.5–28) | Beta(222, 8782) | Warren et al., 2009^38^ |  |
| Colonoscopy with polypectomy (per 1000 colonoscopies) |  |  |  |  |
| Serious GI events | 9.4 (8.2–10.5) | Beta(283, 29850) | Warren et al., 2009^38^ |  |
| Other events | 36.3 (33.3–39.4) | Beta(525, 13933) | Warren et al., 2009^38^ |  |
| Relative risk of complication due to biopsy | 1.22 (1.16–1.30) | Gamma(922, 756) | Chukmaitov et al., 2013^39^ |  |
| Perforation given serious GI events (per 1000 events) | 89.7 | — | Warren et al., 2009^38^ |  |
| Perforation death (per 1000 colonoscopic perforations) | 51.9 | — | Gatto et al., 2003^40^ |  |
| **Disutility** | | | |  |
| Screening procedure |  |  |  |  |
| colonoscopy |  |  |  |  |
| With the test itself | 0.000496 (0.000248–0.000992) | Beta(6, 11396) | Peterse et al. 2021; CISNET document^1,6^ |  |
| With polypectomy / biopsy | 0.001401 (0.000701–0.002802) | Beta(5, 3915) | Peterse et al. 2021; CISNET document^1,6^ |  |
| For cancer diagnosis | 0.001401 (0.000701–0.002802) | Beta(5, 3915) | Peterse et al. 2021; CISNET document^1,6^ |  |
| CTC |  |  |  |  |
| With the test itself | 0.000265 (0.000133–0.000530) | Beta(0.009, 34) | Peterse et al. 2021; CISNET document^1,6^ |  |
| With normal results | 0.000292 (0.000146–0.000584) | Beta(0.84, 2861) | Peterse et al. 2021; CISNET document^1,6^ |  |
| With abnormal results | 0.001559 (0.000780–0.003119) | Beta(0.83, 529) | Peterse et al. 2021; CISNET document^1,6^ |  |
| FIT |  |  |  |  |
| With the test itself | 0.000000 | — | Peterse et al. 2021; CISNET document^1,6^ |  |
| With normal results | 0.000063 (0.000032–0.000127) | Beta(0.009, 143) | Peterse et al. 2021; CISNET document^1,6^ |  |
| With abnormal results | 0.001330 (0.000665–0.002661) | Beta(0.83, 623) | Peterse et al. 2021; CISNET document^1,6^ |  |
| MT-sDNA |  |  |  |  |
| With the test itself | 0.000000 | — | Peterse et al. 2021; CISNET document^1,6^ |  |
| With normal results | 0.000127 (0.000063–0.000253) | Beta(0.009, 72) | Peterse et al. 2021; CISNET document^1,6^ |  |
| With abnormal results | 0.001394 (0.000697–0.002788) | Beta(0.83, 595) | Peterse et al. 2021; CISNET document^1,6^ |  |
| Complications |  |  |  |  |
| Serious GI event | 0.005479 (0.002740–0.010958) | Beta(0.82, 149) | CISNET document^1^ |  |
| Other events | 0.004065 (0.002033–0.008130) | Beta(0.82, 202) | CISNET document^1^ |  |
| Cancer stage |  |  |  |  |
| Stage I |  |  |  |  |
| Initial care | 0.12 | — | CISNET document^1^ |  |
| Continuing care | 0.05 | — | CISNET document^1^ |  |
| Terminal care, death from CRC | 0.70 | — | CISNET document^1^ |  |
| Terminal care, death from other causes | 0.05 | — | CISNET document^1^ |  |
| Stage II |  |  |  |  |
| Initial care | 0.18 | — | CISNET document^1^ |  |
| Continuing care | 0.05 | — | CISNET document^1^ |  |
| Terminal care, death from CRC | 0.70 | — | CISNET document^1^ |  |
| Terminal care, death from other causes | 0.05 | — | CISNET document^1^ |  |
| Stage III |  |  |  |  |
| Initial care | 0.24 | — | CISNET document^1^ |  |
| Continuing care | 0.24 | — | CISNET document^1^ |  |
| Terminal care, death from CRC | 0.70 | — | CISNET document^1^ |  |
| Terminal care, death from other causes | 0.24 | — | CISNET document^1^ |  |
| Stage IV |  |  |  |  |
| Initial care | 0.70 | — | CISNET document^1^ |  |
| Continuing care | 0.70 | — | CISNET document^1^ |  |
| Terminal care, death from CRC | 0.70 | — | CISNET document^1^ |  |
| Terminal care, death from other causes | 0.70 | — | CISNET document^1^ |  |
| **Cost, societal perspective (2022 US dollars)** | | | |  |
| Screening procedure |  |  |  |  |
| Screening colonoscopy w/o polypectomy or biopsy | $2,103 ($1,052–$4,206) | Gamma(alpha=6.8317, theta=307.83) | Medicare fee-for-service; Peterse et al. 2021; calculated^6,42,44,45^ |  |
| Follow-up/diagnostic colonoscopy w/o polypectomy or biopsy | $2,129 ($1,065–$4,259) | Gamma(alpha=6.8274, theta=311.83) | Medicare fee-for-service; Peterse et al. 2021; calculated^6,42,44,45^ |  |
| Surveillance colonoscopy w/o polypectomy or biopsy | $2,103 ($1,051–$4,205) | Gamma(alpha=6.8317, theta=307.83) | Medicare fee-for-service; Peterse et al. 2021; calculated^6,42,44,45^ |  |
| Any colonoscopy with polypectomy or biopsy | $2,524 ($1,262–$5,047) | Gamma(alpha=6.8331, theta=369.38) | Medicare fee-for-service; Peterse et al. 2021; calculated^6,42,44,45^ |  |
| CTC | $735 ($367–$1,469) | Gamma(alpha=6.8357, theta=107.52) | Medicare fee-for-service; Peterse et al. 2021; calculated^6,44,45^ |  |
| FIT | $53 ($27–$107) | Gamma(alpha=6.7444, theta=7.86) | Medicare fee-for-service; Peterse et al. 2021; calculated^6,43^ |  |
| MT-sDNA | $544 ($272–$1,088) | Gamma(alpha=6.8295, theta=79.65) | Medicare fee-for-service; Peterse et al. 2021; calculated^6,43^ |  |
| Complications |  |  |  |  |
| Serious GI event | $17,892 ($8,946–$35,783) | Gamma(alpha=6.83, theta=2619.61) | Peterse et al. 2021^6^ |  |
| Other events | $13,074 ($6,537–$26,149) | Gamma(alpha=6.8288, theta=1914.53) | Peterse et al. 2021^6^ |  |
| Cancer stage |  |  |  |  |
| Stage I |  |  |  |  |
| Initial care | $57,669 ($28,835–$115,338) | Gamma(alpha=6.8296, theta=8443.99) | Peterse et al. 2021^6^ |  |
| Continuing care | $5,741 ($2,871–$11,483) | Gamma(alpha=6.8287, theta=840.71) | Peterse et al. 2021^6^ |  |
| Terminal care, CRC death | $109,030 ($54,515–$218,060) | Gamma(alpha=6.8295, theta=15964.54) | Peterse et al. 2021^6^ |  |
| Terminal care, other death | $35,982 ($17,991–$71,964) | Gamma(alpha=6.8295, theta=5268.61) | Peterse et al. 2021^6^ |  |
| Stage II |  |  |  |  |
| Initial care | $78,191 ($39,096–$156,383) | Gamma(alpha=6.8295, theta=11449.09) | Peterse et al. 2021^6^ |  |
| Continuing care | $6,564 ($3,282–$13,129) | Gamma(alpha=6.8281, theta=961.32) | Peterse et al. 2021^6^ |  |
| Terminal care, CRC death | $121,634 ($60,817–$243,268) | Gamma(alpha=6.8295, theta=17810.06) | Peterse et al. 2021^6^ |  |
| Terminal care, other death | $37,962 ($18,981–$75,924) | Gamma(alpha=6.8295, theta=5558.52) | Peterse et al. 2021^6^ |  |
| Stage III |  |  |  |  |
| Initial care | $109,719 ($54,859–$219,438) | Gamma(alpha=6.8295, theta=16065.52) | Peterse et al. 2021^6^ |  |
| Continuing care | $9,780 ($4,890–$19,560) | Gamma(alpha=6.8295, theta=1432.02) | Peterse et al. 2021^6^ |  |
| Terminal care, CRC death | $126,876 ($63,438–$253,752) | Gamma(alpha=6.8295, theta=18577.61) | Peterse et al. 2021^6^ |  |
| Terminal care, other death | $48,239 ($24,120–$96,478) | Gamma(alpha=6.8296, theta=7063.22) | Peterse et al. 2021^6^ |  |
| Stage IV |  |  |  |  |
| Initial care | $158,872 ($79,436–$317,744) | Gamma(alpha=6.8295, theta=23262.57) | Peterse et al. 2021^6^ |  |
| Continuing care | $43,778 ($21,889–$87,556) | Gamma(alpha=6.8295, theta=6410.12) | Peterse et al. 2021^6^ |  |
| Terminal care, CRC death | $156,979 ($78,489–$313,958) | Gamma(alpha=6.8295, theta=22985.49) | Peterse et al. 2021^6^ |  |
| Terminal care, other death | $102,031 ($51,016–$204,062) | Gamma(alpha=6.8296, theta=14939.62) | Peterse et al. 2021^6^ |  |
| **Other** | | | |  |
| 10-year CRC relative survival by age and stage at diagnosis | **—** | **—** | SEER 17 registries 2000-2009^11^ |  |
| Other-cause mortality | **—** | **—** | 2019 US lifetable^13^ |  |
| Annual discount rate | 3% | — | Weinstein et al. 1996^49^ |  |
| *Abbreviation*: CTC = computed tomographic colonography; FIT = fecal immunochemical test; MT-sDNA = multitarget stool DNA test; GI events = gastrointestinal events; CRC = colorectal cancer | | | |  |
|  |  |  |  |  |

Supporting Table 2. Calibration targets: the disease progression of colorectal cancer (CRC) and CRC screening adherence

| **Type of targets** | **Groups** | **White Men** | **Black Men** | **White Women** | **Black Women** | **Source** |
| --- | --- | --- | --- | --- | --- | --- |
| ***Disease-related targets*** | | | | | | |
| CRC incidence per 100,000 population 2010-2019 | Age 40–44 years | 19 | 18 | 15 | 17 | SEER 17 registries 2010-2019^11^ |
|  | Age 45–49 years | 32 | 35 | 25 | 31 |  |
|  | Age 50–54 years | 58 | 66 | 40 | 51 |  |
|  | Age 55–59 years | 65 | 89 | 42 | 59 |  |
|  | Age 60–64 years | 85 | 118 | 53 | 78 |  |
|  | Age 65–69 years | 108 | 151 | 72 | 103 |  |
|  | Age 70–74 years | 127 | 161 | 93 | 122 |  |
|  | Age 75–79 years | 145 | 169 | 121 | 147 |  |
|  | Age 80–84 years | 173 | 188 | 160 | 171 |  |
| Overall stage at diagnosis, including individuals who ever and never initiated screening (%) | Stage I | 25 | 21 | 24 | 22 | SEER 17 registries 2011-2015^11^ |
|  | Stage II | 26 | 23 | 28 | 24 |  |
|  | Stage III | 27 | 26 | 27 | 27 |  |
|  | Stage IV | 23 | 30 | 22 | 27 |  |
| Stage at diagnosis among individuals who never initiated screening (%) | Stage I | 19 | 17 | 17 | 15 | SEER 9 registries 1975-1979^21^ |
|  | Stage II | 33 | 31 | 35 | 33 |  |
|  | Stage III | 24 | 23 | 25 | 24 |  |
|  | Stage IV | 25 | 30 | 24 | 28 |  |
| Prevalence of adenoma among individuals who never initiated screening (%) | Age 40–44 years | 29 | | 21 | | Rutter et al 2007^8^ |
|  | Age 45–49 years | 33 | | 24 | |  |
|  | Age 50–54 years | 37 | | 27 | |  |
|  | Age 55–59 years | 41 | | 30 | |  |
|  | Age 60–64 years | 45 | | 34 | |  |
|  | Age 65–69 years | 49 | | 37 | |  |
|  | Age 70–74 years | 53 | | 41 | |  |
|  | Age 75–79 years | 57 | | 45 | |  |
|  | Age 80–84 years | 60 | | 48 | |  |
| Adenoma size detected by colonoscopy (%) | Diminutive | 62 | | | | Rutter et al 2009^9^ |
|  | Small | 29 | | | |  |
|  | Large | 9 | | | |  |
| ***Screening utilization targets*** | | | | | | |
| % of population ever had colonoscopy | Age 50–54 years | 43 | 43 | 48 | 42 | NHIS 2010-2019^18^ |
|  | Age 55–59 years | 61 | 51 | 62 | 55 |  |
|  | Age 60–64 years | 71 | 67 | 70 | 61 |  |
|  | Age 65–69 years | 74 | 69 | 73 | 68 |  |
|  | Age 70–74 years | 78 | 75 | 75 | 70 |  |
| % of population ever had stool-based test^†^ | Age 50–54 years | 16 | 16 | 16 | 15 | NHIS 2010-2019^18^ |
|  | Age 55–59 years | 20 | 20 | 23 | 19 |  |
|  | Age 60–64 years | 28 | 28 | 28 | 25 |  |
|  | Age 65–69 years | 31 | 31 | 34 | 27 |  |
|  | Age 70–74 years | 35 | 32 | 36 | 30 |  |
| % of population ever had both coloscopy and stool-based test^†^ | Age 50–54 years | 50 | 48 | 54 | 47 | NHIS 2010-2019^18^ |
|  | Age 55–59 years | 65 | 56 | 68 | 60 |  |
|  | Age 60–64 years | 76 | 70 | 75 | 68 |  |
|  | Age 65–69 years | 78 | 74 | 78 | 73 |  |
|  | Age 70–74 years | 83 | 80 | 80 | 74 |  |
| % of population whose last colonoscopy was within 10 years | Age 50–64 years | 71 | 74 | 68 | 71 | NHIS 2010-2019^18^ |
|  | Age 65–74 years | 70 | 71 | 68 | 71 |  |
| % of population who had stool-based test last year^†^ | Age 50–64 years | 34 | 37 | 33 | 38 | NHIS 2010-2019^18^ |
|  | Age 65–74 years | 32 | 37 | 29 | 36 |  |
| Abbreviation: CRC = colorectal cancer; SEER = Surveillance, Epidemiology, and End Results Program; NHIS = National Health Interview Survey ^†^ Stool-based test was used to parameterize the screening behavior for FIT. | | | | | | |

Supporting Table 3. Calibrated parameters for colorectal cancer (CRC) disease progression and CRC screening adherence

| **Parameters** | **Label** | **Uniform prior distri-bution** | **White Men**  **[95% simulation intervals]** | **Black Men**  **[95% simulation intervals]** | **White Women**  **[95% simulation intervals]** | **Black Women**  **[95% simulation intervals]** |
| --- | --- | --- | --- | --- | --- | --- |
| ***Initial CRC prevalence at age 40*** |  |  |  |  |  |  |
| Initial prevalence of CRC for preclinical cancer stage I | 𝜌_I_ | [0, 0.005] | 0.0005 [0.0000, 0.0022] | 0.0009 [0.0000, 0.0032] | 0.0004 [0.0000, 0.0013] | 0.0005 [0.0000, 0.0017] |
| Multiplicative factor that derives initial prevalence of CRC for stage II, III, and IV | 𝜅 | [0.2, 0.7] | 0.416 [0.205, 0.680] | 0.434 [0.216, 0.680] | 0.437 [0.216, 0.683] | 0.419 [0.209, 0.690] |
| ***Adenoma growth*** |  |  |  |  |  |  |
| Annual rate of developing adenoma |  |  |  |  |  |  |
| Intercept for base rate | 𝛼_0_ | [-7, -4] | -4.5 [-5.3, -4.0] | -4.5 [-5.3, -4.0] | -4.8 [-6.0, -4.1] | -4.7 [-5.8, -4.0] |
| Age effect for age 40-59 years | 𝛼_40_ | [0, 0.1] | 0.042 [0.007, 0.094] | 0.051 [0.006, 0.098] | 0.038 [0.001, 0.091] | 0.044 [0.004, 0.097] |
| Age effect for age 60-69 years | 𝛼_60_ | [0, 0.2] | 0.076 [0.004, 0.192] | 0.083 [0.002, 0.187] | 0.070 [0.006, 0.164] | 0.072 [0.003, 0.183] |
| Age effect for age ≥70 years | 𝛼_70_ | [0, 0.2] | 0.082 [0.005, 0.191] | 0.082 [0.003, 0.187] | 0.075 [0.006, 0.196] | 0.075 [0.003, 0.181] |
| Annual adenoma growth rate |  |  |  |  |  |  |
| Intercept for base rate | β_0_ | [-7, -3] | -4.7 [-5.1, -4.4] | -4.3 [-4.7, -4.0] | -5.0 [-5.5, -4.5] | -4.4 [-4.8, -4.1] |
| Age effect | β_age_ | [0, 0.1] | 0.019 [0.000, 0.042] | 0.016 [0.000, 0.037] | 0.022 [0.002, 0.046] | 0.014 [0.000, 0.034] |
| Duration effect | β_dur_ | [0, 0.1] | 0.036 [0.007, 0.059] | 0.033 [0.006, 0.057] | 0.038 [0.006, 0.067] | 0.036 [0.010, 0.055] |
| Effect for small adenoma | β_small_ | [0, 0.1] | 0.054 [0.004, 0.098] | 0.051 [0.001, 0.098] | 0.049 [0.002, 0.098] | 0.056 [0.005, 0.099] |
| Effect for large adenoma | β_large_ | [0, 0.1] | 0.044 [0.003, 0.094] | 0.044 [0.002, 0.098] | 0.050 [0.004, 0.097] | 0.046 [0.002, 0.096] |
| ***CRC progression rate*** |  |  |  |  |  |  |
| Preclinical CRC stage I to stage II |  |  |  |  |  |  |
| Intercept for base rate | 𝜆_10_ | [-10, 2] | -6.1 [-9.1, -2.4] | -6.1 [-9.9, -2.4] | -6.5 [-9.4, -2.5] | -5.6 [-9.7, -2.2] |
| Age effect | 𝜆_11_ | [0, 0.2] | 0.135 [0.035, 0.199] | 0.123 [0.020, 0.197] | 0.139 [0.046, 0.196] | 0.124 [0.022, 0.195] |
| Duration effect | 𝜆_12_ | [0, 0.2] | 0.097 [0.004, 0.196] | 0.109 [0.004, 0.200] | 0.103 [0.010, 0.190] | 0.107 [0.006, 0.196] |
| Preclinical CRC stage II to stage III |  |  |  |  |  |  |
| Intercept for base rate | 𝜆_20_ | [-10, 2] | -6.2 [-9.8, -2.5] | -6.7 [-9.9, -2.3] | -6.3 [-10.0, -2.1] | -6.9 [-9.9, -2.5] |
| Age effect | 𝜆_21_ | [0, 0.2] | 0.108 [0.030, 0.188] | 0.122 [0.019, 0.198] | 0.102 [0.022, 0.175] | 0.125 [0.032, 0.192] |
| Duration effect | 𝜆_22_ | [0, 0.2] | 0.106 [0.005, 0.193] | 0.109 [0.006, 0.195] | 0.092 [0.004, 0.196] | 0.093 [0.000, 0.194] |
| Preclinical CRC stage III to stage IV |  |  |  |  |  |  |
| Intercept for base rate | 𝜆_30_ | [-10, 2] | -6.7 [-9.8, -2.1] | -6.7 [-9.8, -2.4] | -6.5 [-9.9, -2.2] | -7.2 [-10.0, -2.5] |
| Age effect | 𝜆_31_ | [0, 0.2] | 0.100 [0.018, 0.159] | 0.105 [0.021, 0.175] | 0.093 [0.022, 0.152] | 0.109 [0.025, 0.159] |
| Duration effect | 𝜆_32_ | [0, 0.2] | 0.095 [0.004, 0.195] | 0.095 [0.004, 0.195] | 0.107 [0.007, 0.187] | 0.096 [0.005, 0.190] |
| ***Incidental CRC detection*** |  |  |  |  |  |  |
| Intercept for base rate | 𝛿_0_ | [-7, 0] | -3.8 [-6.7, -0.2] | -4.9 [-6.9, -0.7] | -4.0 [-6.9, -0.6] | -4.1 [-6.9, -0.3] |
| Age effect | 𝛿_age_ | [0, 0.3] | 0.118 [0.004, 0.293] | 0.073 [0.000, 0.272] | 0.143 [0.004, 0.290] | 0.109 [0.005, 0.284] |
| Duration effect by stage |  |  |  |  |  |  |
| Stage I | 𝛿_I_ | [0, 1] | 0.491 [0.030, 0.957] | 0.413 [0.002, 0.958] | 0.511 [0.024, 0.972] | 0.501 [0.021, 0.968] |
| Stage II | 𝛿_II_ | [0, 1] | 0.480 [0.067, 0.980] | 0.398 [0.033, 0.968] | 0.502 [0.017, 0.982] | 0.487 [0.043, 0.999] |
| Stage III | 𝛿_III_ | [0, 0.3] | 0.159 [0.020, 0.290] | 0.168 [0.014, 0.289] | 0.151 [0.009, 0.292] | 0.125 [0.003, 0.277] |
| Stage IV | 𝛿_IV_ | [0, 0.3] | 0.151 [0.008, 0.295] | 0.145 [0.007, 0.288] | 0.150 [0.016, 0.292] | 0.138 [0.010, 0.289] |
| ***Screening adherence*** |  |  |  |  |  |  |
| Screening colonoscopy initiation |  |  |  |  |  |  |
| Initiating screening colonoscopy at age 50-54 | 𝜁_C50_ | [0, 0.1] | 0.081 [0.054, 0.098] | 0.063 [0.028, 0.098] | 0.081 [0.052, 0.100] | 0.070 [0.042, 0.096] |
| Multiplicative factor for age 55-59 | 𝜅_C55_ | [0, 1] | 0.525 [0.067, 0.963] | 0.511 [0.042, 0.951] | 0.485 [0.035, 0.982] | 0.555 [0.086, 0.984] |
| Multiplicative factor for age 60-64 | 𝜅_C60_ | [0, 1] | 0.577 [0.071, 0.982] | 0.648 [0.075, 0.977] | 0.423 [0.018, 0.971] | 0.514 [0.032, 0.981] |
| Multiplicative factor for age 65-69 | 𝜅_C65_ | [0, 1] | 0.534 [0.035, 0.991] | 0.599 [0.052, 0.980] | 0.493 [0.038, 0.974] | 0.562 [0.046, 0.972] |
| Multiplicative factor for age 70-75 | 𝜅_C70_ | [0, 1] | 0.560 [0.047, 0.965] | 0.575 [0.024, 0.980] | 0.499 [0.027, 0.979] | 0.543 [0.024, 0.961] |
| Adherence to subsequent screening colonoscopy intervals |  |  |  |  |  |  |
| Adherence at age ≥65 | 𝛾_C65_ |  | 0.565 [0.077, 0.959] | 0.647 [0.214, 0.978] | 0.566 [0.176, 0.942] | 0.587 [0.106, 0.981] |
| Multiplicative factor for age 50-64 | 𝜏_C50_ | [0, 0.1] | 0.423 [0.008, 0.967] | 0.312 [0.001, 0.821] | 0.537 [0.061, 0.976] | 0.444 [0.033, 0.968] |
| Average subsequent screening colonoscopy adherence (calculated) |  |  | 0.402 | 0.425 | 0.435 | 0.423 |
| Probability of switching from colonoscopy to FIT | 𝜉_CF_ | [0, 1] | 0.343 [0.055, 0.766] | 0.326 [0.056, 0.700] | 0.300 [0.033, 0.638] | 0.338 [0.048, 0.749] |
| Screening FIT initiation |  |  |  |  |  |  |
| Initiating screening FIT at age 50-54 | 𝜁_F50_ | [0, 0.1] | 0.031 [0.001, 0.079] | 0.026 [0.002, 0.066] | 0.035 [0.002, 0.081] | 0.020 [0.000, 0.056] |
| Multiplicative factor for age 55-59 | 𝜅_F55_ | [0, 1] | 0.507 [0.038, 0.958] | 0.479 [0.020, 0.967] | 0.466 [0.039, 0.952] | 0.445 [0.019, 0.963] |
| Multiplicative factor for age 60-64 | 𝜅_F60_ | [0, 1] | 0.515 [0.045, 0.992] | 0.517 [0.027, 0.970] | 0.446 [0.016, 0.932] | 0.469 [0.026, 0.968] |
| Multiplicative factor for age 65-69 | 𝜅_F65_ | [0, 1] | 0.481 [0.011, 0.975] | 0.576 [0.050, 0.965] | 0.537 [0.034, 0.999] | 0.507 [0.047, 0.984] |
| Multiplicative factor for age 70-75 | 𝜅_F70_ | [0, 1] | 0.473 [0.019, 0.991] | 0.534 [0.024, 0.943] | 0.538 [0.021, 0.972] | 0.544 [0.019, 0.972] |
| Adherence to subsequent screening FIT intervals |  |  |  |  |  |  |
| Adherence at age ≥65 | 𝛾_F65_ |  | 0.561 [0.210, 0.932] | 0.587 [0.197, 0.931] | 0.616 [0.167, 0.969] | 0.641 [0.233, 0.965] |
| Multiplicative factor for age 50-64 | 𝜏_F50_ | [0, 0.1] | 0.500 [0.053, 0.973] | 0.484 [0.017, 0.992] | 0.523 [0.016, 0.975] | 0.530 [0.015, 0.973] |
| Average subsequent screening FIT adherence (calculated) |  |  | 0.420 | 0.436 | 0.469 | 0.490 |
| Probability of switching from FIT to colonoscopy | 𝜉_FC_ | [0, 1] | 0.141 [0.037, 0.290] | 0.129 [0.026, 0.280] | 0.163 [0.042, 0.285] | 0.140 [0.031, 0.280] |
| Abbreviation: CRC = colorectal cancer; FIT = fecal immunochemical test | | | | | | |

Supporting Table 4. Medicare payment rate or cost for medical resource utilization for screening, diagnostic, and surveillance procedures by HCPCS / CPT code

| HCPCS / CPT code | Description | Type of fee schedule and components for calculation | | | Cost |
| --- | --- | --- | --- | --- | --- |
| Screening colonoscopy without polypectomy or biopsy | | | | | |
| HCPCS / CPT code | Description | Physician fee (facility price)^a^ | Outpatient prospective payment system^b^ | | Total payment rate |
| G0121 | Colonoscopy on individual not meeting criteria for high risk | $181.75 | $871.71 | | $1,053.46 |
| Follow-up colonoscopy without polypectomy or biopsy | | | | | |
| HCPCS / CPT code | Description | Physician fee (facility price)^a^ | Outpatient prospective payment system^b^ | | Total payment rate |
| 45378 | Diagnostic colonoscopy | $181.42 | $871.71 | | $1,053.13 |
| Surveillance colonoscopy without polypectomy or biopsy | | | | | |
| HCPCS / CPT code | Description | Physician fee (facility price)^a^ | Outpatient prospective payment system^b^ | | Total payment rate |
| G0105 | Colonoscopy on individual at high risk | $181.42 | $871.71 | | $1,053.13 |
| Any colonoscopy with polypectomy or biopsy | | | | | |
| HCPCS / CPT code | Description | Physician fee (facility price)^a^ | Outpatient prospective payment system^b^ | Total frequency^b^ | Total payment rate |
| 45380 | Colonoscopy and biopsy | $197.06 | $1,125.53 | 401203 | $1,322.59 |
| 45381 | Colonoscopy with directed submucosal injection(s), any substance | $196.73 | $1,125.53 | 32493 | $1,322.26 |
| 45382 | Colonoscopy with control of bleeding, any method | $253.65 | $1,125.53 | 5814 | $1,379.18 |
| 45384 | Colonoscopy with lesion removal by hot biopsy forceps | $224.36 | $1,125.53 | 20172 | $1,349.89 |
| 45385 | Colonoscopy with lesion removal by snare technique (usually used to perform polypectomy) | $249.32 | $1,125.53 | 425030 | $1,374.85 |
| Pathologist | | | | | |
| HCPCS / CPT code |  | Physician fee (non-facility price)^a^ | | | Total payment rate |
| 88305 | Level IV surgical pathology, gross and microscopic examination | $71.57 | | | $71.57 |
| Computed tomography colonography (CTC)^c^ | | | | | |
| HCPCS / CPT code | Description | Outpatient prospective payment system^b^ | | Total frequency^b^ | Total payment rate |
| 74261 | Diagnostic CTC without IV contrast | $218.37 | | 3971 | $218.37 |
| 74262 | Diagnostic CTC with IV contrast | $294.59 | | 362 | $294.59 |
| Fecal immunochemical test | | | | | |
| HCPCS / CPT code | Description | Laboratory diagnostic fee schedule^d^ | | | Total payment rate |
| G0328 | Colorectal cancer screening; fecal occult blood test, immunoassay, 1-3 simultaneous | $18.05 | | | $18.05 |
| Multitarget stool DNA test (MT-sDNA) | | | | | |
| HCPCS / CPT code | Description | Laboratory diagnostic fee schedule^d^ | | | Total payment rate |
| 81528 | Oncology (colorectal) screening, quantitative real-time target and signal amplification of 10 dna markers | $508.87 | | | $508.87 |
| Anesthesia^e^ | | | | | |
| HCPCS / CPT code | Description | National anesthesia conversion factor | Time units | Anesthesia base units | Total payment rate |
| 00811 | Anesthesia for diagnostic colonoscopy | 20.4349 | 1.7 | 4 | $116.48 |
| 00812 | Anesthesia for screening colonoscopy | 20.4349 | 1.4 | 3 | $89.91 |
| Pharmaceutical price^f^ | | | | | |
| HCPCS / CPT code | Description | Pharmaceutical price from the federal supply schedule | | | Estimated cost |
| NA | Bowel preparation^g^ | $110.00 | | | $110.00 |
| NA | Anesthesia agent^h^ | $6.49 | | | $6.49 |
| ^a^ Data on for physician fee were from 2024 Physician Fee Schedule from: https://www.cms.gov/medicare/physician-fee-schedule/search. We assumed that all these procedures were done in hospital facility.  ^b^ Data on 2024 Medicare payment rate and number of claims for hospital resource utilization were based on the Outpatient Prospective Payment System (OPPS) <https://www.cms.gov/license/ama?file=/files/zip/2024-nfrm-opps-cost-statistics-files.zip>. The total frequency was used to calculate the weighted estimate across multiple HCPCS / CPT codes.  ^c^ Because screening CTC is not covered by Medicare, the estimate was based on diagnostic CTC with and without contrast, which only included the OPPS payment rate but not physician fee.  ^d^ Data on the cost for FIT and MT-sDNA tests were from the 2024 Clinical Laboratory Fee Schedule from: <https://www.cms.gov/license/ama?file=/files/zip/24CLABQ2.zip>  ^e^ Cost for anesthesia was estimated based on the 2024 information for anesthesiologist from: <https://www.cms.gov/center/provider-type/anesthesiologists-center>. Fee for anesthesiologist was calculated as (Base Units + Time [in units]) x CF.  ^f^ Data on pharmaceutical prices were from the Veteran Affairs Federal Supply Schedule: https://www.va.gov/opal/nac/fss/pharmprices.asp ^g^ We based our estimate on SUPREP® BOWEL PREP KIT.  ^h^ The cost of anesthesia agent was parameterized using propofol with an average dosage of 200 mg. The dosage estimate was based on Childer et al. 2015.^48^ | | | | | |

Supporting Table 5. Societal costs of screening, diagnostic, and surveillance procedures (2024 US $)

|  | Medicare payment rate^a^ | |  |  |  |  |  |
| --- | --- | --- | --- | --- | --- | --- | --- |
| Procedure | Procedure | Anesthesia | Total CMS payment | Bowel prep | Patient / escort time^b^ (hours) | Time cost^c^ | Total cost (2024 US dollar) |
| Screening colonoscopy w/o polypectomy or biopsy | $1,053 | $96 | $1,150 | $110 | 24 | $843 | $2,103 |
| Diagnostic colonoscopy w/o polypectomy or biopsy | $1,053 | $123 | $1,176 | $110 | 24 | $843 | $2,129 |
| Surveillance colonoscopy w/o polypectomy or biopsy | $1,053 | $96 | $1,150 | $110 | 24 | $843 | $2,103 |
| colonoscopy with polypectomy or biopsy^d^ | $1,447 | $123 | $1,570 | $110 | 24 | $843 | $2,524 |
| CTC | $225 | NA | $225 | $110 | 11 | $400 | $735 |
| FIT | $18 | NA | $18 | NA | 1 | $35 | $53 |
| MT-sDNA | $509 | NA | $509 | NA | 1 | $35 | $544 |
| CTC: CT colonography; FIT: fecal immunochemical test; MT-sDNA: multitarget stool DNA test; NA: not available  *Note:* Table was constructed following Peterse et al. 2021^6^ with updated cost estimates.  ^a^ Payment rate for a procedure included physician fee and fee for hospital outpatient service; payment rate for anesthesia was from anesthesia fee-for-service.  ^b^ Only colonoscopy related procedures included both patient and escort time but not the other procedures. The estimates for patient or escort time were based on Peterse et al. 2021, which excluded sleep time (two nights for colonoscopy and one night for CTC).  ^c^ Time cost was estimated using patient / escort time multiplied by $35.24, which is 2023 average hourly wage (from the Economic Report of the President, accessed on 6/23/2024) inflated to 2024 US $.  ^d^ The payment rate for colonoscopy with polypectomy or biopsy was a weighted average across CPT codes 45380, 45381, 45382, 45384, and 45385 by the total frequency reported in the cost statistics from the 2024 Medicare hospital outpatient prospective payment system. | | | | | | | |
|  |  |  |  |  |  |  |  |

Supporting Table 6. Disutility associated with screening, diagnostic, and surveillance procedure

| Procedure | Disutility^a^ | Patient time^b^ | Utility loss converted to annual scale | Source |
| --- | --- | --- | --- | --- |
| *Utility loss due to screening procedure itself* | | | | |
| Colonoscopy | 0.12 | 36.21 hours | 0.000496 | Peterse et al. 2021; CISNET document^1,6^ |
| CTC | 0.12 | 19.35 hours | 0.000265 | Peterse et al. 2021; CISNET document^1,6^ |
| FIT | 0 |  | 0 | Peterse et al. 2021; CISNET document^1,6^ |
| MT-sDNA | 0 |  | 0 | Peterse et al. 2021; CISNET document^1,6^ |
| *Utility loss due to waiting for test results* | | | | |
| Colonoscopy w/o polypectomy or biopsy | 0 | 0 days | 0 | Peterse et al. 2021; CISNET document^1,6^ |
| Colonoscopy with polypectomy or biopsy | 0.033036 | 10 days | 0.000905 | Peterse et al. 2021; CISNET document^1,6^ |
| CTC | 0.003304 | 3 days | 0.000027 | Peterse et al. 2021; CISNET document^1,6^ |
| FIT | 0.003304 | 7 days | 0.000063 | Peterse et al. 2021; CISNET document^1,6^ |
| MT-sDNA | 0.003304 | 14 days | 0.000127 | Peterse et al. 2021; CISNET document^1,6^ |
| *Utility loss due to waiting for follow-up colonoscopy* | | | | |
| CTC | 0.033036 | 14 days | 0.001267 | Peterse et al. 2021; CISNET document^1,6^ |
| FIT | 0.033036 | 14 days | 0.001267 | Peterse et al. 2021; CISNET document^1,6^ |
| MT-sDNA | 0.033036 | 14 days | 0.001267 | Peterse et al. 2021; CISNET document^1,6^ |
| CTC: computed tomographic colonography; FIT: fecal immunochemical test; MT-sDNA: multitarget stool DNA test ^a^ Disutility estimates were derived from the CISNET Colorectal Cancer Work Group technical report for the USPSTF (Knudsen et al. 2021).  ^b^ Data on patient time were from Peterse et al. 2021 and the CISNET Colorectal Cancer Work Group technical report (Knudsen et al. 2021). We excluded escort time and accounted for the sleep time in patient time. | | | | |

Supporting Table 7. Comparison of adenoma dwell time and preclinical cancer sojourn time between our model and three CISNET models: SimCRC, CRC-SPIN, and MISCAN

| Model | Estimates | | | |
| --- | --- | --- | --- | --- |
| Adenoma dwell time (years)^§^ | | | | |
| CISNET models^*^ | Overall [interquartile range] | | | |
| SimCRC | 21.2 | | | |
|  | [12–29] | | | |
| CRC-SPIN | 25.4 | | | |
|  | [16–33] | | | |
| MISCAN | 12.5 | | | |
|  | [4–18] | | | |
| Our model | White Men | Black Men | White Women | Black Women |
|  | 32.6 | 30.7 | 34.3 | 33.2 |
| Preclinical cancer sojourn time (years)^¶^ | | | | |
| CISNET models^*^ | Overall [interquartile range] | | | |
| SimCRC | 4.0 | | | |
|  | [2–5] | | | |
| CRC-SPIN | 3.6 | | | |
|  | [2–5] | | | |
| MISCAN | 4.7 | | | |
|  | [1–7] | | | |
| Our model | White Men | Black Men | White Women | Black Women |
|  | 3.6 | 4.2 | 3.5 | 3.7 |
| * Simulated outcomes extracted from the CISNET model technical report ^1^ | | | | |
| § Adenoma dwell time was calculated as the duration from adenoma onset to the onset of  preclinical cancer stage I. | | | | |
| ¶ Preclinical cancer sojourn time was calculated as the duration between the onset of preclinical cancer to the time at cancer diagnosis | | | | |

Supporting Table 8. Comparison of CRC incidence per 1000 40-year-old adults in the absence of screening between our model and three CISNET models: SimCRC, CRC-SPIN, and MISCAN

| Model | White Men | Black Men | White Women | Black Women |
| --- | --- | --- | --- | --- |
| CISNET models* |  |  |  |  |
| SimCRC | 92 | 78 | 78 | 72 |
| CRC-SPIN | 80 | 68 | 74 | 68 |
| MISCAN | 87 | 74 | 77 | 71 |
| Our model  [95% simulation range] | 84 [65 – 110] | 67 [55 – 82] | 82 [65 – 103] | 78 [62 – 94] |
| * Simulated outcomes from the CISNET model technical report^1^ | | | | |

Supporting Table 9. Cost-effectiveness analysis under perfect screening, diagnostic, and surveillance adherence by race and gender

| Strategies | Number of CRC cases per 1000 population | Number of screening tests per 1000 population | Number of non-colonoscopy screening tests per 1000 population | Number of screening colonoscopies per 1000 population | Number of all colonoscopies per 1000 population | QALYG per 1000 population | Cost per 1000 population (million $) | Incremental cost-effectiveness ratio |  |
| --- | --- | --- | --- | --- | --- | --- | --- | --- | --- |
| White Men | | | | | | | | |  |
| No screening | 84 | — | — | — | — | 0 | $8.10 | — |  |
| Screening strategies |  |  |  |  |  |  |  |  |  |
| FIT-only | 11 | 23,679 | 23,679 | 0 | 1,687 | 97 | $4.59 | Cost-effective |  |
| CTC-only | 11 | 4,746 | 4,746 | 0 | 1,452 | 96 | $5.61 | Dominated |  |
| Colonoscopy-only | 4 | 2,544 | 0 | 2,544 | 3,179 | 103 | $6.00 | $251,091 |  |
| MT-sDNA-only | 16 | 8,126 | 8,126 | 0 | 1,543 | 87 | $6.83 | Dominated |  |
| Black Men | | | | | | | | |  |
| No screening | 67 | — | — | — | — | 0 | $6.86 | — |  |
| Screening strategies |  |  |  |  |  |  |  |  |  |
| FIT-only | 12 | 21,982 | 21,982 | 0 | 1,542 | 91 | $4.58 | Cost-effective |  |
| CTC-only | 10 | 4,457 | 4,457 | 0 | 1,347 | 92 | $5.42 | Dominated |  |
| Colonoscopy-only | 6 | 2,425 | 0 | 2,425 | 3,006 | 96 | $6.04 | $273,008 |  |
| MT-sDNA-only | 15 | 7,592 | 7,592 | 0 | 1,416 | 82 | $6.63 | Dominated |  |
| White Women | | | | | | | | |  |
| No screening | 82 | — | — | — | — | 0 | $7.21 | — |  |
| Screening strategies |  |  |  |  |  |  |  |  |  |
| FIT-only | 10 | 25,697 | 25,697 | 0 | 1,582 | 83 | $4.28 | Cost-effective |  |
| CTC-only | 14 | 5,139 | 5,139 | 0 | 1,321 | 78 | $5.64 | Dominated |  |
| Colonoscopy-only | 3 | 2,695 | 0 | 2,695 | 3,182 | 88 | $5.83 | $318,444 |  |
| MT-sDNA-only | 16 | 8,736 | 8,736 | 0 | 1,455 | 72 | $6.65 | Dominated |  |
| Black Women | | | | | | | | |  |
| No screening | 78 | — | — | — | — | 0 | $7.49 | — |  |
| Screening strategies |  |  |  |  |  |  |  |  |  |
| FIT-only | 11 | 24,548 | 24,548 | 0 | 1,522 | 102 | $4.28 | Cost-effective |  |
| CTC-only | 12 | 4,940 | 4,940 | 0 | 1,291 | 100 | $5.46 | Dominated |  |
| Colonoscopy-only | 4 | 2,617 | 0 | 2,617 | 3,093 | 109 | $5.80 | $234,658 |  |
| MT-sDNA-only | 15 | 8,377 | 8,377 | 0 | 1,406 | 90 | $6.61 | Dominated |  |
| Abbreviations: CRC = colorectal cancer; QALYG = quality-adjusted life-years gained relative to no screening; CTC = CT colonography; FIT = fecal immunochemical test; MT-sDNA = multitarget stool DNA test | | | | | | | | |  |
|  |  |  |  |  |  |  |  |  |  |

Supporting Figures

**
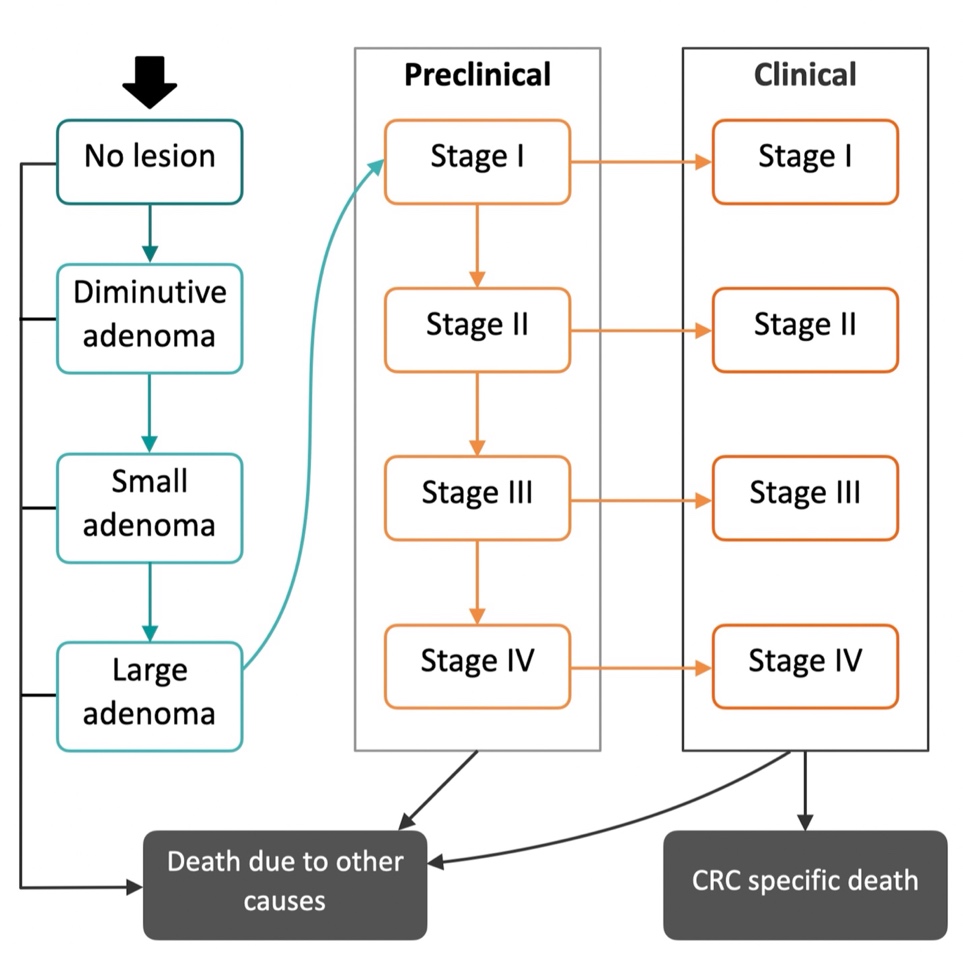
**

Supporting Figure 1. State-transition diagram for the natural history of colorectal cancer in the absence of screening


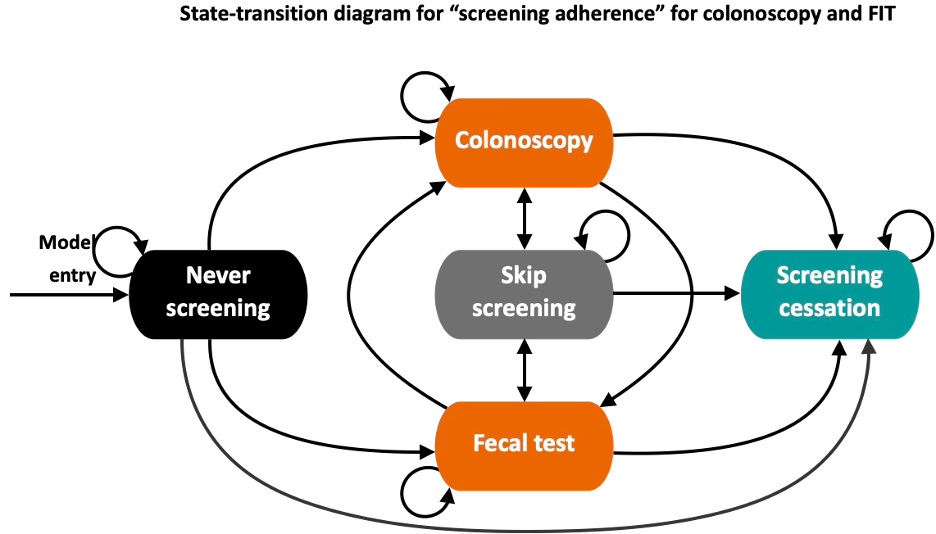


Supporting Figure 2. State-transition diagram for screening adherence for colonoscopy and FIT in the baseline model.

| (A) Prevalence of adenoma among individuals who never initiated screening  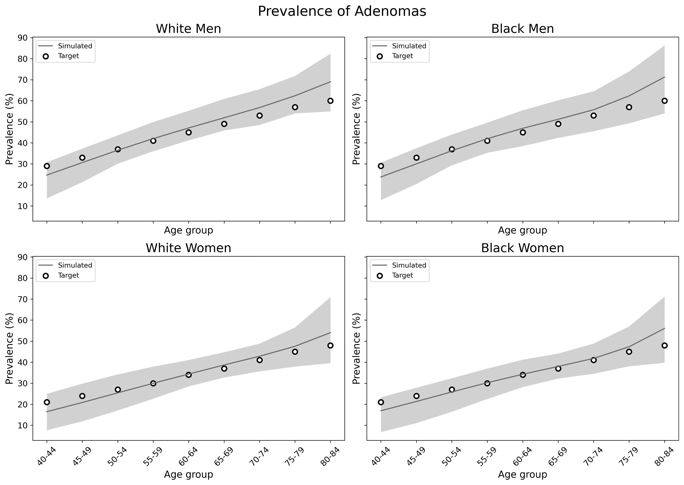 | 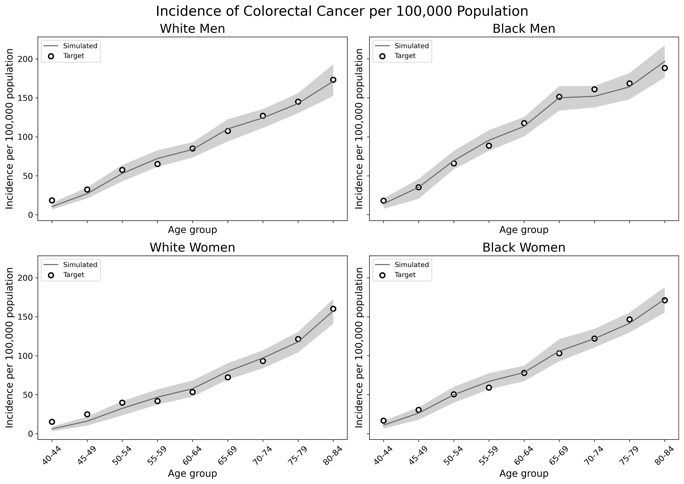(B) CRC incidence per 100,000 population with screening colonoscopy and FIT available in the population (SEER data: 2010-2019) |
| --- | --- |
| 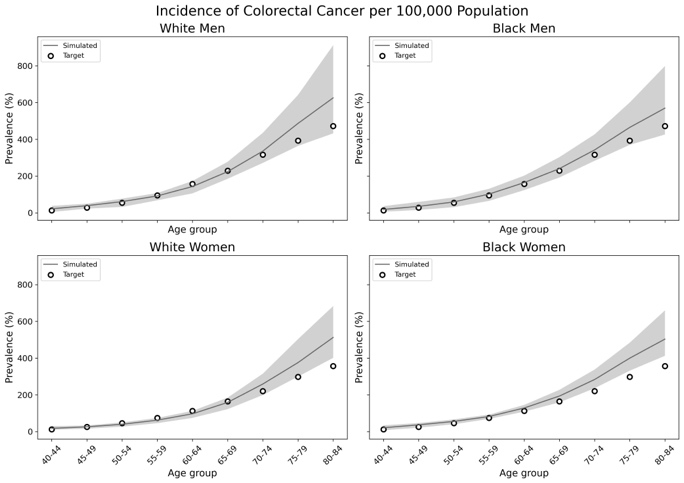(C) CRC incidence per 100,000 population in the absence of screening compared to the 1975-1979 SEER data | 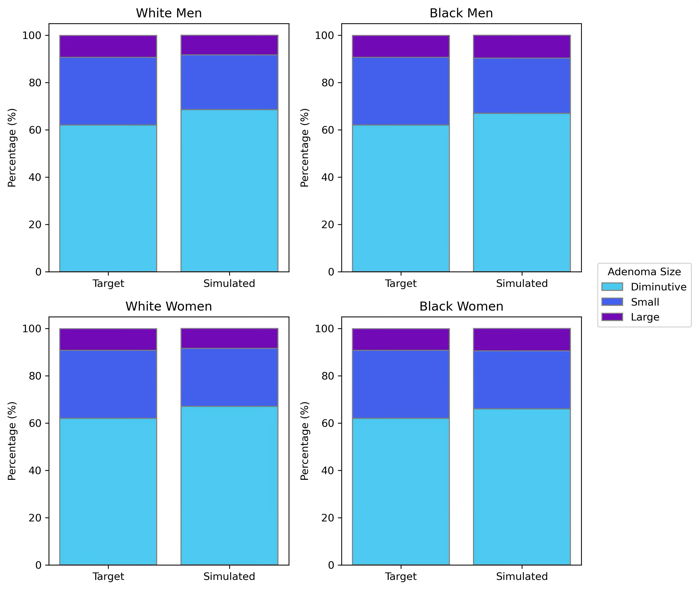(D) Distribution of adenoma size detected by colonoscopy |

Supplementary Figure 3. Simulated disease-related outcomes compared to calibration targets by type of target: (A) prevalence of adenoma among individuals who never sought screening; (B) CRC incidence per 100,000 population with screening colonoscopy and FIT available in the population; (C) CRC incidence per 100,000 population in the absence of screening compared to the 1975-1979 SEER data^§^; (D) Distribution of adenoma size detected by colonoscopy

^§^ CRC incidence from 1975-1979 SEER data was not used for calibration due to documented increases in CRC risk in the U.S. population over time.^50^ This figure provides a comparative reference between simulated and historical CRC incidence in the absence of screening.


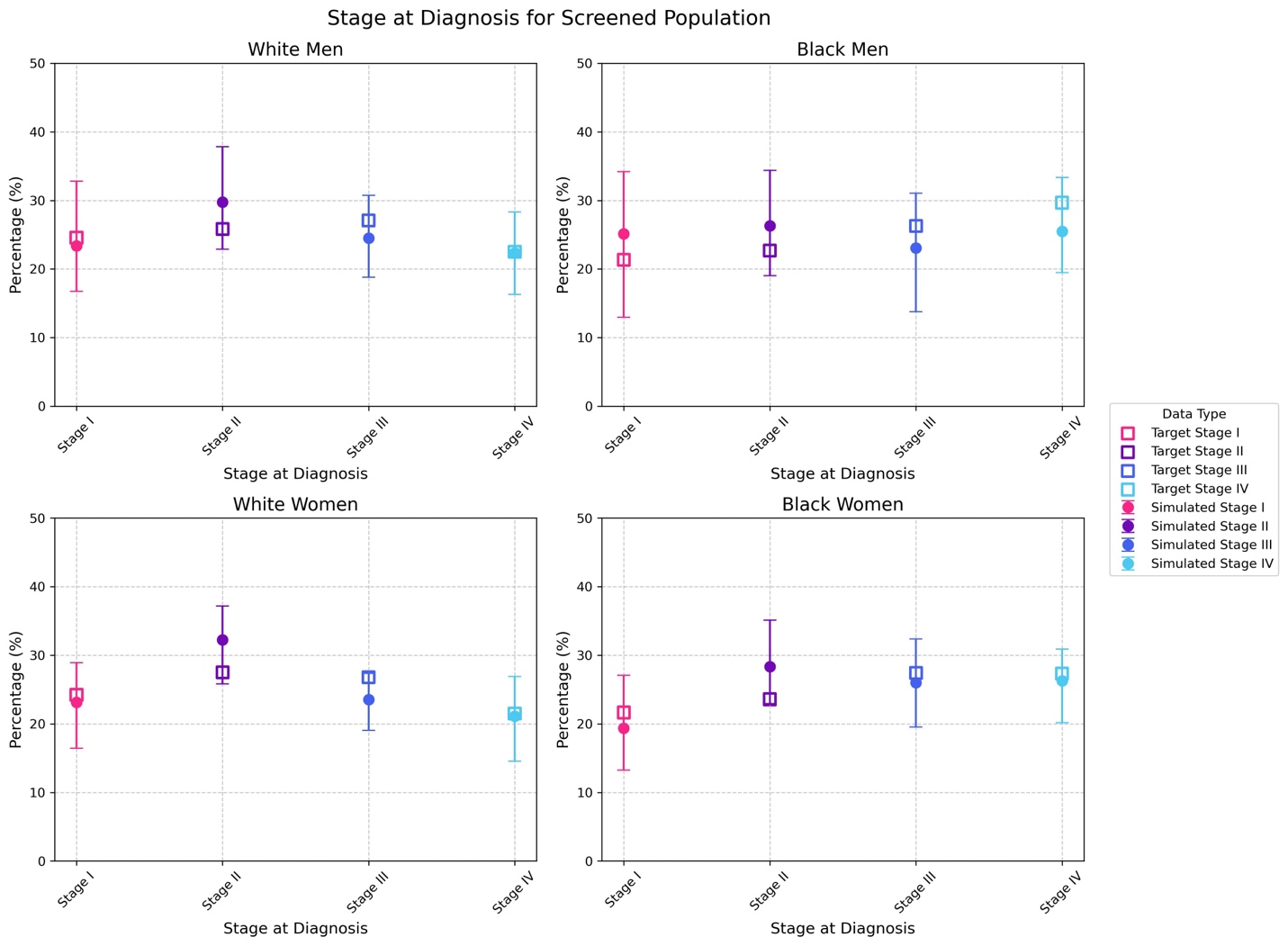


Supporting Figure 4. Simulated stage at diagnosis (both individuals who ever and never had screening) compared to the targets from 2010-2015 SEER data. Solid circles denoted simulated means; vertical intervals represent 95% ranges of the simulated outcomes; and open squares are calibration targets


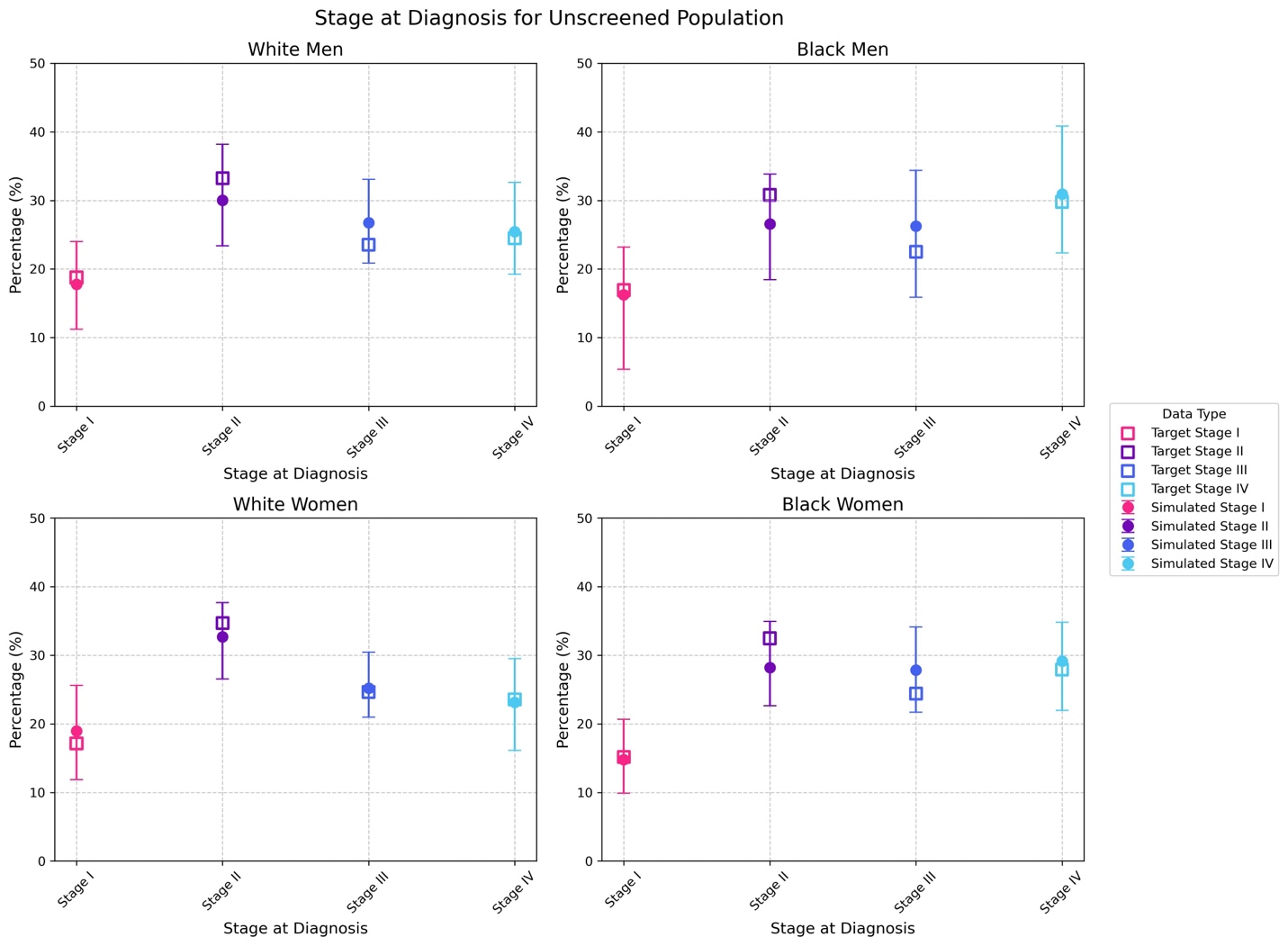


Supporting Figure 5. Simulated stage at diagnosis among individuals who never initiated screening compared to the targets from 1975-1979 SEER data. Solid circles denoted simulated means; vertical intervals represent 95% ranges of the simulated outcomes; and open squares are calibration targets

| (A) Proportion of population who ever had 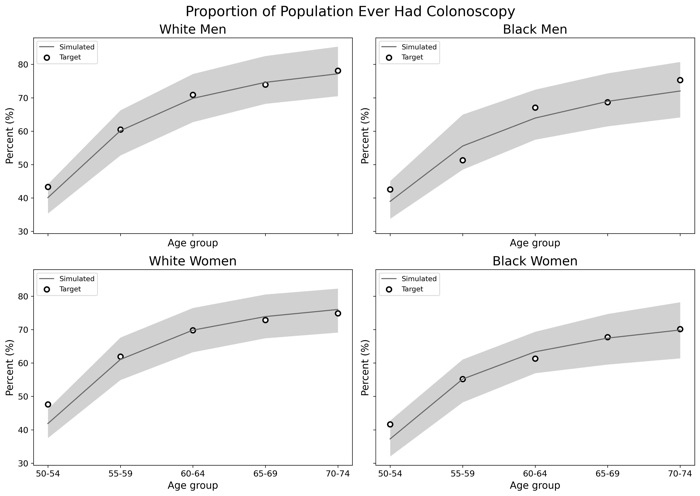colonoscopy | (B) Proportion of population who ever had stool-based test (assumed to be FIT)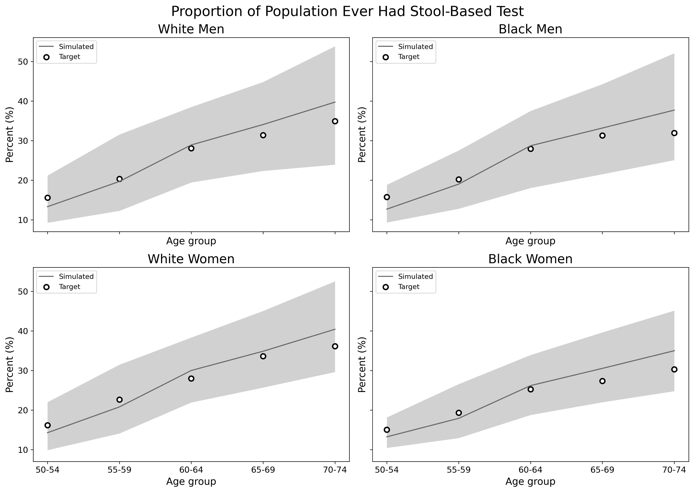 |
| --- | --- |
| 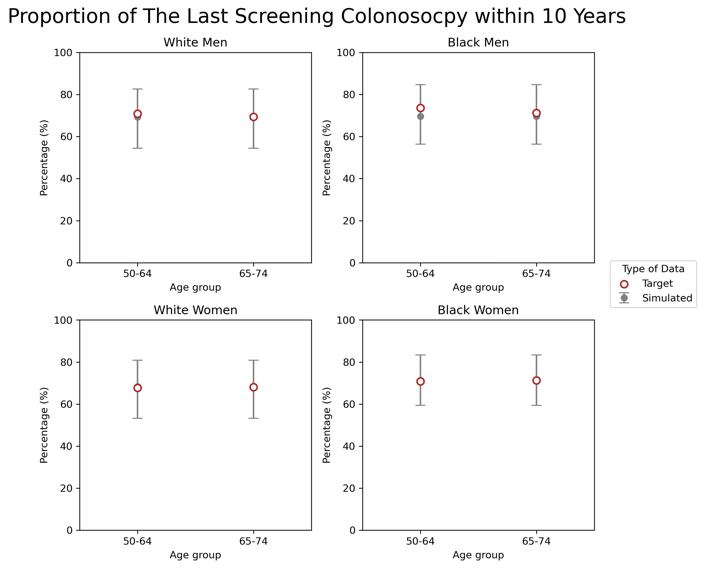(C) Proportion of individuals undergoing screening colonoscopy within 10 years among those who ever started colonoscopy | 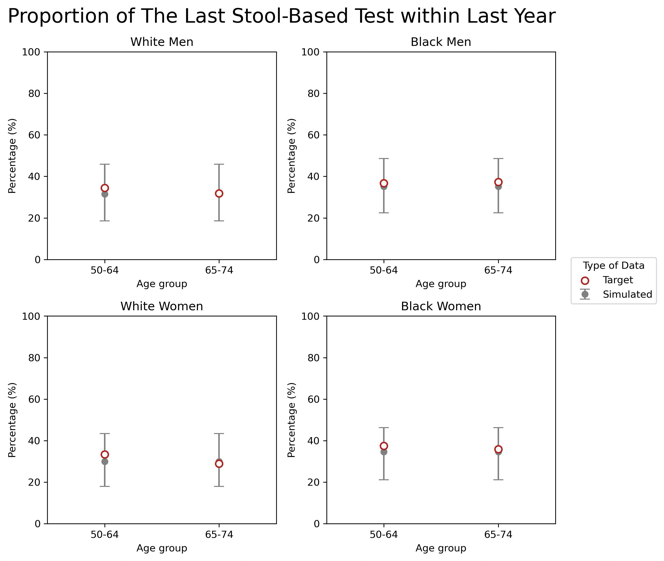(D) Proportion of individuals undergoing FIT within last year among those who ever started FIT |

Supporting Figure 6. Simulated screening behavior-related outcomes compared to the 2010-2019 NHIS calibration targets by type of target: (A) proportion of population who ever started colonoscopy; (B) proportion of population who ever started stool-based test (assumed to be FIT); (C) proportion of individuals undergoing screening colonoscopy within 10 years among those who ever started colonoscopy; (D) proportion of individuals undergoing FIT within last year among those who ever started FIT

eReferences

1. Knudsen AB, Rutter CM, Peterse EFP, et al. *Colorectal Cancer Screening: An Updated Decision Analysis for the U.S. Preventive Services Task Force*. Agency for Healthcare Research and Quality (US); 2021. Accessed September 8, 2022. http://www.ncbi.nlm.nih.gov/books/NBK570833/

2. Knudsen AB, Lansdorp-Vogelaar I, Rutter CM, et al. Cost-Effectiveness of Computed Tomographic Colonography Screening for Colorectal Cancer in the Medicare Population. *JNCI: Journal of the National Cancer Institute*. 2010;102(16):1238-1252. doi:10.1093/jnci/djq242

3. Knudsen AB, Rutter CM, Peterse EFP, et al. Colorectal Cancer Screening: An Updated Modeling Study for the US Preventive Services Task Force. *JAMA*. 2021;325(19):1998. doi:10.1001/jama.2021.5746

4. Knudsen AB, Zauber AG, Rutter CM, et al. Estimation of Benefits, Burden, and Harms of Colorectal Cancer Screening Strategies: Modeling Study for the US Preventive Services Task Force. *JAMA*. 2016;315(23):2595. doi:10.1001/jama.2016.6828

5. Meester RGS, Peterse EFP, Knudsen AB, et al. Optimizing colorectal cancer screening by race and sex: Microsimulation analysis II to inform the American Cancer Society colorectal cancer screening guideline: Modeling CRC Screening by Race and Sex. *Cancer*. 2018;124(14):2974-2985. doi:10.1002/cncr.31542

6. Peterse EFP, Meester RGS, de Jonge L, et al. Comparing the Cost-Effectiveness of Innovative Colorectal Cancer Screening Tests. *JNCI: Journal of the National Cancer Institute*. 2021;113(2):154-161. doi:10.1093/jnci/djaa103

7. Peterse EFP, Meester RGS, Siegel RL, et al. The impact of the rising colorectal cancer incidence in young adults on the optimal age to start screening: Microsimulation analysis I to inform the American Cancer Society colorectal cancer screening guideline: Young-Onset CRC: Screening Implications. *Cancer*. 2018;124(14):2964-2973. doi:10.1002/cncr.31543

8. Rutter CM, Yu O, Miglioretti DL. A hierarchical non-homogenous Poisson model for meta-analysis of adenoma counts. *Statist Med*. 2007;26(1):98-109. doi:10.1002/sim.2460

9. Rutter CM, Miglioretti DL, Savarino JE. Bayesian Calibration of Microsimulation Models. *Journal of the American Statistical Association*. 2009;104(488):1338-1350. doi:10.1198/jasa.2009.ap07466

10. Rutter CM, Ozik J, DeYoreo M, Collier N. Microsimulation model calibration using incremental mixture approximate Bayesian computation. *Ann Appl Stat*. 2019;13(4). doi:10.1214/19-AOAS1279

11. Surveillance, Epidemiology, and End Results (SEER) Program (www.seer.cancer.gov) SEER*Stat Database: Incidence - SEER Research Data, 17 Registries, Nov 2023 Sub (1975-2021) - Linked To County Attributes - Time Dependent (1990-2022) Income/Rurality, 1969-2022 Counties, National Cancer Institute, DCCPS, Surveillance Research Program, released April 2024, based on the November 2023 submission. Accessed January 15, 2024. www.seer.cancer.gov

12. Sawicki T, Ruszkowska M, Danielewicz A, Niedźwiedzka E, Arłukowicz T, Przybyłowicz KE. A Review of Colorectal Cancer in Terms of Epidemiology, Risk Factors, Development, Symptoms and Diagnosis. *Cancers*. 2021;13(9):2025. doi:10.3390/cancers13092025

13. Arias E, Xu J. United States Life Tables, 2019. *Natl Vital Stat Rep*. 2022;70(19):1-59.

14. Townsley RM, Koutouan PR, Mayorga ME, Mills SD, Davis MM, Hasmiller Lich K. When History and Heterogeneity Matter: A Tutorial on the Impact of Markov Model Specifications in the Context of Colorectal Cancer Screening. *Med Decis Making*. 2022;42(7):845-860. doi:10.1177/0272989X221097386

15. US Preventive Services Task Force, Bibbins-Domingo K, Grossman DC, et al. Screening for Colorectal Cancer: US Preventive Services Task Force Recommendation Statement. *JAMA*. 2016;315(23):2564. doi:10.1001/jama.2016.5989

16. Cronin KA, Mariotto AB, Clarke LD, Feuer EJ. Chapter 5: Additional Common Inputs for Analyzing Impact of Adjuvant Therapy and Mammography on U.S. Mortality. *JNCI Monographs*. 2006;2006(36):26-29. doi:10.1093/jncimonographs/lgj005

17. Cronin KA, Yu B, Krapcho M, et al. Modeling the dissemination of mammography in the United States. *Cancer Causes Control*. 2005;16(6):701-712. doi:10.1007/s10552-005-0693-8

18. National Center for Health Statistics. National Health Interview Survey, 2010, 2013, 2015, 2018, 2019. Published online 2024. https://www.cdc.gov/nchs/nhis/index.htm

19. Ebner DW, Finney Rutten LJ, Miller-Wilson LA, et al. Trends in Colorectal Cancer Screening from the National Health Interview Survey: Analysis of the Impact of Different Modalities on Overall Screening Rates. *Cancer Prevention Research*. 2024;17(6):275-280. doi:10.1158/1940-6207.CAPR-23-0443

20. Hassmiller Lich K, O’Leary MC, Nambiar S, et al. Estimating the impact of insurance expansion on colorectal cancer and related costs in North Carolina: A population-level simulation analysis. *Preventive Medicine*. 2019;129:105847. doi:10.1016/j.ypmed.2019.105847

21. Surveillance, Epidemiology, and End Results (SEER) Program (www.seer.cancer.gov) SEER*Stat Database: Incidence - SEER Research Data, 9 Registries, Nov 2020 Sub (1975-2018) - Linked To County Attributes - Time Dependent (1990-2018) Income/Rurality, 1969-2019 Counties, National Cancer Institute, DCCPS, Surveillance Research Program, released April 2021, based on the November 2020 submission. Accessed January 15, 2024. www.seer.cancer.gov

22. Bergstra J, Bardenet R, Bengio Y, Kégl B. Algorithms for Hyper-Parameter Optimization. In: ; 2011.

23. Krivorotko O, Sosnovskaia M, Vashchenko I, Kerr C, Lesnic D. Agent-based modeling of COVID-19 outbreaks for New York state and UK: Parameter identification algorithm. *Infectious Disease Modelling*. 2022;7(1):30-44. doi:10.1016/j.idm.2021.11.004

24. Kuntz KM, Lansdorp-Vogelaar I, Rutter CM, et al. A Systematic Comparison of Microsimulation Models of Colorectal Cancer: The Role of Assumptions about Adenoma Progression. *Med Decis Making*. 2011;31(4):530-539. doi:10.1177/0272989X11408730

25. Kunst N, Alarid-Escudero F, Aas E, Coupé VMH, Schrag D, Kuntz KM. Estimating Population-Based Recurrence Rates of Colorectal Cancer over Time in the United States. *Cancer Epidemiology, Biomarkers & Prevention*. 2020;29(12):2710-2718. doi:10.1158/1055-9965.EPI-20-0490

26. van Rijn JC, Reitsma JB, Stoker J, Bossuyt PM, van Deventer SJ, Dekker E. Polyp Miss Rate Determined by Tandem Colonoscopy: A Systematic Review. *Am J Gastroenterology*. 2006;101(2):343-350. doi:10.1111/j.1572-0241.2006.00390.x

27. de Haan MC, van Gelder RE, Graser A, Bipat S, Stoker J. Diagnostic value of CT-colonography as compared to colonoscopy in an asymptomatic screening population: a meta-analysis. *Eur Radiol*. 2011;21(8):1747-1763. doi:10.1007/s00330-011-2104-8

28. Pickhardt PJ, Hassan C, Halligan S, Marmo R. Colorectal Cancer: CT Colonography and Colonoscopy for Detection—Systematic Review and Meta-Analysis. *Radiology*. 2011;259(2):393-405. doi:10.1148/radiol.11101887

29. Imperiale TF, Ransohoff DF, Itzkowitz SH, et al. Multitarget Stool DNA Testing for Colorectal-Cancer Screening. *N Engl J Med*. 2014;370(14):1287-1297. doi:10.1056/NEJMoa1311194

30. Mohl JT, Ciemins EL, Miller-Wilson LA, Gillen A, Luo R, Colangelo F. Rates of Follow-up Colonoscopy After a Positive Stool-Based Screening Test Result for Colorectal Cancer Among Health Care Organizations in the US, 2017-2020. *JAMA Netw Open*. 2023;6(1):e2251384. doi:10.1001/jamanetworkopen.2022.51384

31. Sali L, Mascalchi M, Falchini M, et al. Reduced and Full-Preparation CT Colonography, Fecal Immunochemical Test, and Colonoscopy for Population Screening of Colorectal Cancer: A Randomized Trial. *JNCIJ*. 2016;108(2):djv319. doi:10.1093/jnci/djv319

32. Lieberman DA, Rex DK, Winawer SJ, Giardiello FM, Johnson DA, Levin TR. Guidelines for Colonoscopy Surveillance After Screening and Polypectomy: A Consensus Update by the US Multi-Society Task Force on Colorectal Cancer. *Gastroenterology*. 2012;143(3):844-857. doi:10.1053/j.gastro.2012.06.001

33. Summers RM. Polyp Size Measurement at CT Colonography: What Do We Know and What Do We Need to Know? *Radiology*. 2010;255(3):707-720. doi:10.1148/radiol.10090877

34. US Preventive Services Task Force, Davidson KW, Barry MJ, et al. Screening for Colorectal Cancer: US Preventive Services Task Force Recommendation Statement. *JAMA*. 2021;325(19):1965. doi:10.1001/jama.2021.6238

35. van Hees F, Habbema JDF, Meester RG, Lansdorp-Vogelaar I, van Ballegooijen M, Zauber AG. Should Colorectal Cancer Screening Be Considered in Elderly Persons Without Previous Screening?: A Cost-Effectiveness Analysis. *Ann Intern Med*. 2014;160(11):750. doi:10.7326/M13-2263

36. Djinbachian R, Dubé AJ, Durand M, et al. Adherence to post-polypectomy surveillance guidelines: a systematic review and meta-analysis. *Endoscopy*. 2019;51(07):673-683. doi:10.1055/a-0865-2082

37. Gupta S, Lieberman D, Anderson JC, et al. Recommendations for Follow-Up After Colonoscopy and Polypectomy: A Consensus Update by the US Multi-Society Task Force on Colorectal Cancer. *Gastroenterology*. 2020;158(4):1131-1153.e5. doi:10.1053/j.gastro.2019.10.026

38. Warren JL, Klabunde CN, Mariotto AB, et al. Adverse events after outpatient colonoscopy in the Medicare population. *Ann Intern Med*. 2009;150(12):849-857, W152. doi:10.7326/0003-4819-150-12-200906160-00008

39. Chukmaitov A, Bradley CJ, Dahman B, Siangphoe U, Warren JL, Klabunde CN. Association of polypectomy techniques, endoscopist volume, and facility type with colonoscopy complications. *Gastrointestinal Endoscopy*. 2013;77(3):436-446. doi:10.1016/j.gie.2012.11.012

40. Gatto NM, Frucht H, Sundararajan V, Jacobson JS, Grann VR, Neugut AI. Risk of Perforation After Colonoscopy and Sigmoidoscopy: A Population-Based Study. *JNCI Journal of the National Cancer Institute*. 2003;95(3):230-236. doi:10.1093/jnci/95.3.230

41. van Hees F, Zauber AG, Klabunde CN, Goede SL, Lansdorp-Vogelaar I, van Ballegooijen M. The Appropriateness of More Intensive Colonoscopy Screening Than Recommended in Medicare Beneficiaries: A Modeling Study. *JAMA Intern Med*. 2014;174(10):1568. doi:10.1001/jamainternmed.2014.3889

42. Centers for Medicare Medicaid Services. Anesthesiologists Center. 2022. Accessed February 21, 2023. https://www.cms.gov/Center/Provider-Type/Anesthesiologists-Center

43. Centers for Medicare Medicaid Services. Clinical Laboratory Fee Schedule. 2022. Accessed February 21, 2023. https://www.cms.gov/medicare/medicare-fee-for-service-payment/clinicallabfeesched

44. Centers for Medicare Medicaid Services. Hospital Outpatient PPS. 2024. Accessed June 10, 2024. https://www.cms.gov/medicare/medicare-fee-for-service-payment/hospitaloutpatientpps

45. Centers for Medicare Medicaid Services. Physician fee schedule look-up tool. 2024. Accessed June 10, 2024. https://www.cms.gov/medicare/physician-fee-schedule/search/overview

46. Council of Economic Advisers. *Economic Report of the President, Transmitted to the Congress March 2023 Together with the Annual Report of the Council of Economic Advisers*. Council of Economic Advisers; 2023.

47. U.S. Department of Veterans Affairs. Pharmaceutical Prices. 2023. Accessed February 21, 2024. https://www.va.gov/opal/nac/fss/pharmprices.asp

48. Childers RE, Williams JL, Sonnenberg A. Practice patterns of sedation for colonoscopy. *Gastrointestinal Endoscopy*. 2015;82(3):503-511. doi:10.1016/j.gie.2015.01.041

49. Weinstein MC, Siegel JE, Gold MR, Kamlet MS, Russel LB. Recommendations of the Panel on Cost-Efectiveness in Health and Medicine. *JAMA*. 1996;276(15):6.

50. Siegel RL, Fedewa SA, Anderson WF, et al. Colorectal Cancer Incidence Patterns in the United States, 1974–2013. *JNCI: Journal of the National Cancer Institute*. 2017;109(8). doi:10.1093/jnci/djw322
